# Computational Analysis and Prediction of the Binding Motif and Protein Interacting Partners of the Abl SH3 Domain

**DOI:** 10.1371/journal.pcbi.0020001

**Published:** 2006-01-27

**Authors:** Tingjun Hou, Ken Chen, William A McLaughlin, Benzhuo Lu, Wei Wang

**Affiliations:** 1 Department of Chemistry and Biochemistry, University of California San Diego, La Jolla, California, United States of America; 2 Center for Theoretical Biological Physics, University of California San Diego, La Jolla, California, United States of America; Cornell University, United States of America

## Abstract

Protein-protein interactions, particularly weak and transient ones, are often mediated by peptide recognition domains, such as Src Homology 2 and 3 (SH2 and SH3) domains, which bind to specific sequence and structural motifs. It is important but challenging to determine the binding specificity of these domains accurately and to predict their physiological interacting partners. In this study, the interactions between 35 peptide ligands (15 binders and 20 non-binders) and the Abl SH3 domain were analyzed using molecular dynamics simulation and the Molecular Mechanics/Poisson-Boltzmann Solvent Area method. The calculated binding free energies correlated well with the rank order of the binding peptides and clearly distinguished binders from non-binders. Free energy component analysis revealed that the van der Waals interactions dictate the binding strength of peptides, whereas the binding specificity is determined by the electrostatic interaction and the polar contribution of desolvation. The binding motif of the Abl SH3 domain was then determined by a virtual mutagenesis method, which mutates the residue at each position of the template peptide relative to all other 19 amino acids and calculates the binding free energy difference between the template and the mutated peptides using the Molecular Mechanics/Poisson-Boltzmann Solvent Area method. A single position mutation free energy profile was thus established and used as a scoring matrix to search peptides recognized by the Abl SH3 domain in the human genome. Our approach successfully picked ten out of 13 experimentally determined binding partners of the Abl SH3 domain among the top 600 candidates from the 218,540 decapeptides with the PXXP motif in the SWISS-PROT database. We expect that this physical-principle based method can be applied to other protein domains as well.

## Synopsis

One of the central questions of molecular biology is to understand how signals are transduced in the cell. Intracellular signal transduction is mainly achieved through cascades of protein-protein interactions, which are often mediated by peptide-binding modular domains, such as Src Homology 2 and 3 (SH2 and SH3). Each family of these domains binds to peptides with specific sequence and structural characteristics. To reconstruct the protein-protein interaction networks mediated by modular domains, one must identify the peptide motifs recognized by these domains and understand the mechanism of binding specificity. These questions are challenging because the domain-peptide interactions are usually weak and transient. Here, the authors took a physical-principles approach to address these difficult questions for the SH3 domain of human protein Abl, which binds to peptides containing the PXXP motif (where P is proline and X is any amino acid). They generated a position-specific scoring matrix to represent the binding motif of the Abl SH3 domain. Analysis on the binding free energy components suggested insights into how the binding specificity is achieved. Most known protein interacting partners of the Abl SH3 domain were correctly identified using the position-specific scoring matrix, and other potential interacting partners were also suggested.

## Introduction

The interactions between protein domains and their peptide ligands play critical roles in signal transduction and many other key biological processes. Because domain-peptide interactions are usually weak and transient, and often depend upon post-translational modification, they tend to be under-represented in high-throughput and computational studies [1], thus highlighting the need to develop new methods to identify these interactions. The Src Homology 3 (SH3) domain is the most abundant modular domain in the human proteome and presents in a wide variety of proteins, such as kinases, lipases, GTPases, and adaptor proteins, to orchestrate diverse cellular processes [2–6]. SH3 domains are 50–70 amino acids long and consist of five β-strands arranged into two sheets packed at right angles. They recognize the proline-rich peptides with the consensus motif PXXP (where P is proline and X is any amino acid) [7, 8] that forms a left-handed poly-proline type II (PPII) helix [9]. Depending on the position of the positive residue in the peptide sequence, the majority of SH3 ligands fall into two classes that bind to the protein in opposite orientations [10]: N-terminal to C-terminal (class I) or C-terminal to N-terminal (class II). Class I peptides typically consist of a core motif of RXLPX#P (where # is usually a hydrophobic residue), whereas the class II peptides contain a core motif of PX#PXR. In class I peptides, the proline residues in bold occupy the sites in the hydrophobic pocket that are normally referred as position P_0_ and P_3_, while the Arg residue occupies position P_−3_ (the positions are often dubbed as P_−3_, P_−2_, P_−1_, P_0_, P_1_, P_2,_ and P_3_ from N-terminal to C-terminal, namely from R to P, in the motif RXLPX#P [2]). A notable variance of this motif is the one recognized by the Abl SH3 domain, which contains a tyrosine or a large hydrophobic residue at position P_−3_.

The binding specificity of a specific SH3 domain is determined by the flanking residues around the core motif PXXP [10]. Understanding the molecular basis of the specificity for each SH3 domain and identifying the sequence motif it recognizes are crucial to reconstruct the complete protein-protein interaction networks mediated by SH3 domains. Both experimental and computational methods have been developed to tackle this problem. Peptide library screening is often used to determine the binding motif of a SH3 domain, in which the binding peptides are sequenced and aligned to generate a frequency matrix representing the amino acid preference at each position [11, 12]. Bias may be introduced by not completely sampling all possible peptides, not quantitatively weighting the contribution of peptides to the matrix based on their binding strength and/or not distinguishing peptides bound to the SH3 domain in different binding modes. Computational methods such as Scansite [13], SPOT [14], and VIP [15] methods have been developed to predict interacting proteins of a domain. The performance of Scansite totally depends on the accuracy of the frequency matrix determined by the peptide library experiments. SPOT is limited by the relatively small number of residue contact pairs between SH3 domains and peptides. The performance of the VIP method can be improved if more conformational sampling is done and more rigorous binding energy prediction method, including the conformational energy change and the desolvation contribution upon peptide binding, is applied.

In this study, we analyzed the binding specificity of the SH3 domain of the human protein Abl. The Molecular Mechanics/Poisson-Boltzmann Solvent Area (MM/PBSA) method [16] was first applied to calculate the binding free energies between the Abl SH3 domains and 35 ten-residue-long peptides (15 binders and 20 non-binders). As a validation of the MM/PBSA method on the domain-peptide system, the calculated binding free energies of the 15 known binders correlated well with the experimental values [17] and were distinct from those of the non-binders. Analysis of the molecular dynamics (MD) trajectories and binding free energy components shed light into understanding the mechanism of the binding specificity of the Abl SH3 domain. The residue preference at each position of the peptide ligand was then studied systematically by single position mutation and MM/PBSA calculations, which we call the virtual mutagenesis (VM) method [18, 19]. A single position mutation free energy profile (SPMFEP) was established from such analysis to quantitatively represent the binding motif and was in good agreement with the experimental measurements. We used SPMFEP as a scoring matrix to search the SWISS-PROT database for potential binding partners of the Abl SH3 domain. Most experimentally determined binding proteins of the Abl SH3 domain were ranked in the top 600 candidates among about 6.2 × 10^7^ decapeptides in the database and many promising candidates were also suggested.

## Results

### Molecular Basis of the Binding Specificity of the Abl SH3 Domain

#### The calculated binding free energies correlate well with the experimental values.

**Figure 1:**
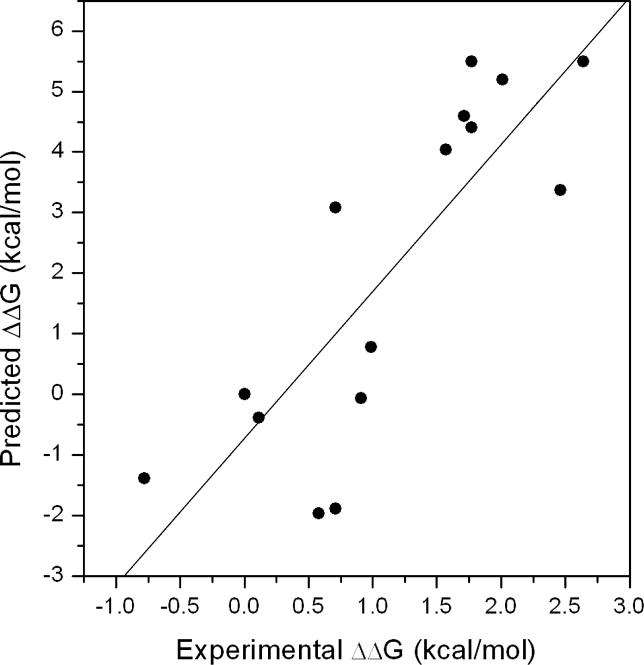
The Correlations between Experimental and Predicted Relative Binding Free Energies of the 15 Binders of the Abl SH3 Domain

We first evaluated the performance of the MM/PBSA method on calculating the binding free energies of the Abl SH3 domain and its peptide ligands (15 binders and 20 non-binders). As shown in Table 1 and Figure 1, the calculated relative binding free energies of the 15 known binders show good correlation with the experimental values (the correlation coefficient *r* and standard deviation [SD] are 0.82 and 1.7, respectively). We then analyzed the free energy components to search for the dominant factor that dictates the binding specificity (Table 1). As the favorable electrostatic interaction between the peptide and the SH3 domain Δ*E*_ele_ is canceled by the unfavorable electrostatic contribution to desolvation Δ*G*_PB_, the van der Waals interaction Δ*E*_vdw_ is the most favorable component of the binding free energy. The favorable Δ*E*_vdw_ is mainly from the interactions between the conserved proline residues of the PXXP motif and the hydrophobic surface that is formed by Tyr7, Phe9, Trp36, Tyr52, and Pro49 of the Abl SH3 domain and conserved in almost all SH3 domains. To investigate which energetic factor determines the relative binding affinities of these 15 binders, we compared the correlations between the measured binding free energies and each of the four free energy components, Δ*E*_ele_, Δ*E*_vdw_, Δ*G*_PB_, and Δ*G*_SA_. None of these components shows good correlation with the experimental values and the largest correlation coefficient is only 0.43 for Δ*E*_ele_, which suggests that no individual free energy component dominates the binding specificity. We then analyzed the non-polar (Δ*E*_vdw_ + Δ*G*_SA_) and the electrostatic contribution (Δ*E*_ele_ + Δ*G*_PB_) to the binding free energy. The electrostatic contribution correlates well with the binding free energies (*r* = 0.73, SD = 1.6) while the non-polar contribution does not show any correlation (*r* = −0.32, SD = 2.0). It suggests that the binding preference of these peptides is mainly determined by the electrostatic contribution to binding.

**Table 1:**
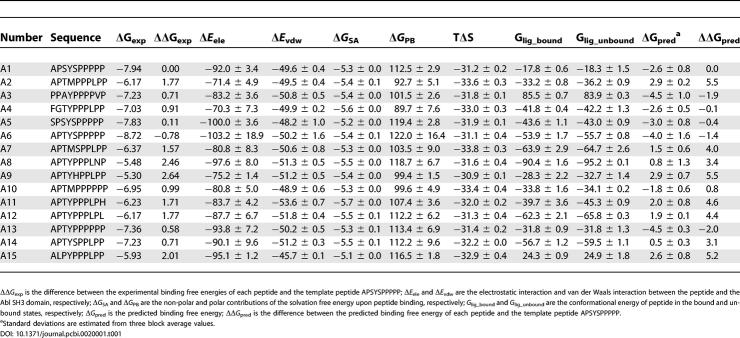
Energetic Components and Binding Affinities for the 15 Peptide Ligands of the Abl SH3 Domain (kcal/mol)

We also found that the conformational change of the peptide upon binding Δ*E*_conf_pep_ was important (*r* = 0.54, SD =1.8). The sum of Δ*E*_ele_, Δ*E*_vdw_, Δ*G*_PB_, Δ*G*_SA_, and Δ*E*_conf_pep_ correlates well with the binding free energies (*r* = 0.79, SD =1.8) whereas the sum of the first four terms does not (*r* = 0.52). Our calculations highlight the crucial effect of the change of the conformational energy of peptide to the affinities, which is not always appropriately considered [20, 21]. Inclusion of the conformational entropy −TΔS in the free energy calculation only slightly improves the correlation coefficient from 0.79–0.82, which suggests that the conformational entropy is not the determinant factor of the binding specificity of the binders.

**Figure 2:**
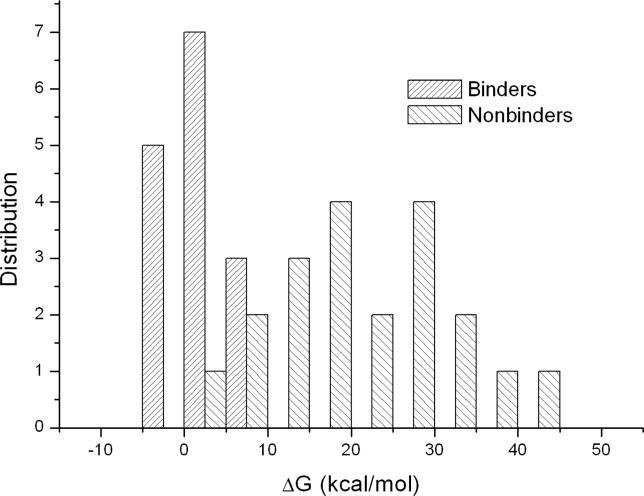
The Distributions of the Predicted Binding Free Energies for Binders and Non-Binders of the Abl SH3 Domain

The binding free energies and the free energy components for the 20 non-binders of the Abl SH3 domain were also calculated (Table S1). Two distributions of the binding free energies for binders and non-binders are distinct (Figure 2), which indicates that the MM/PBSA method can distinguish binders from non-binders. We also found that most non-binders preferred unbound conformations. First, the average conformational entropy change upon binding −TΔS for binders and non-binders are 32.1 kcal/mol and 35.9 kcal/mol, respectively, indicating that most non-binders lost more entropy upon binding than did binders. Second, the change of conformational energy for binders and non-binders are also significantly different: the average value for binders and non-binders are 2.0 kcal/mol and 4.3 kcal/mol, respectively.

#### The binding motif can be revealed by the VM method.

To understand the mechanism of the binding specificity, we need to determine the binding motif of the domain. We analyzed the amino acid preference at each position of the peptide using the VM method (see Materials and Methods). We compared our results with the available experimental measurements at positions P_3_, P_0_, P_−3,_ and P_−5_ (Tables S3–S6) [17, 22]. These four positions are particularly important for the peptide binding: the two usually conserved Pro residues at P_3_ and P_0_ ensure strong binding affinity and residues at P_−3_, and P_−5_ are essential to the binding specificity [9, 10]. To determine the residues of the Abl SH3 domain that are important for peptide binding, the contribution of each SH3 domain residue to binding with the template peptide APSYSP**P**PP**P** (the two conserved Pro residues are in bold) was analyzed (the polar contribution to desolvation was calculated using the GB/SA method implemented in the *mm_pbsa* module of AMBER 8 for the sake of efficiency.) (Table S7) [19].

**Figure 3:**
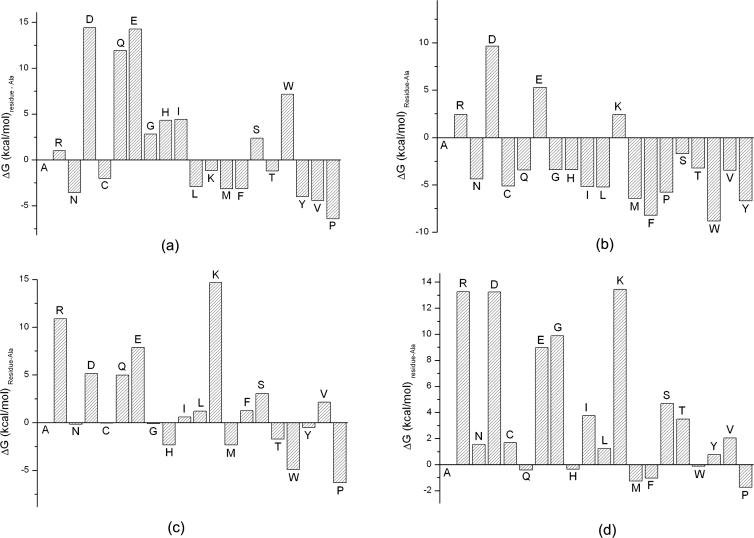
The Preference of Residue Based on the Binding Free Energy Difference between the Mutated Peptide and the Template Peptide The preference of residue at positions (A) P_−5_, (B) P_−3_, (C) P_0_, and (D) P_3_ based on the binding free energy difference between the mutated peptide and the template peptide APSYSPPPPP. In (D), the conformational entropy was included in the binding free energies.

In the crystal structure of APSYSPPPPP complexed with the Abl SH3 domain, the residue at position P_−5_ in the peptide occupies a hydrophobic pocket of the SH3 domain. Proline at this position has relatively strong van der Waals interactions with Trp36 (−1.3 kcal/mol) and Trp47 (−0.9 kcal/mol) in the Abl SH3 domain (Table S7). Several other residues, Asn, Leu, Met, Phe, Tyr, and Val, are also strongly favored at this position (Figure 3A). It is worth pointing out that the mutation of Pro to Tyr or Phe, does not significantly impair the van der Waals interaction between the peptide and the SH3 domain due to the conformational change of the peptide backbone. Our predicted preference based on free energy calculations is consistent with the experimental results: Pro is the most preferred, whereas other residues especially hydrophobic ones (Phe, Leu, Met, Val, and Trp) are also favored [22].

It should be noted that the preference of residue at P_−5_ are closely related to the residues at the adjacent positions. For example, the known binder FGTYPPPLPP (A4 in Table 1) has a Gly at P_−5_, which is not favored at this position based on the VM result. By analyzing the MD trajectory on A4-SH3 complex, we found that Phe at P_−6_ in A4 can occupy the binding pocket that is occupied by Pro at P_−5_ in the template peptide APSYSPPPPP to form favorable van der Waals interactions with Trp36 and Trp47. Moreover, the benzyl ring of Phe at P_−6_ is parallel to the aromatic ring of Trp47 to form strong π-π stacking interactions. Therefore, if there is a small residue (Ala, Gly, or Ser) at P_−5_, an aromatic residue (Tyr, Phe, or Trp) may be preferred at P_−6_. This suggests that the repertoire of SH3 domain-binding peptides may be much larger than previously thought.

**Figure 4:**
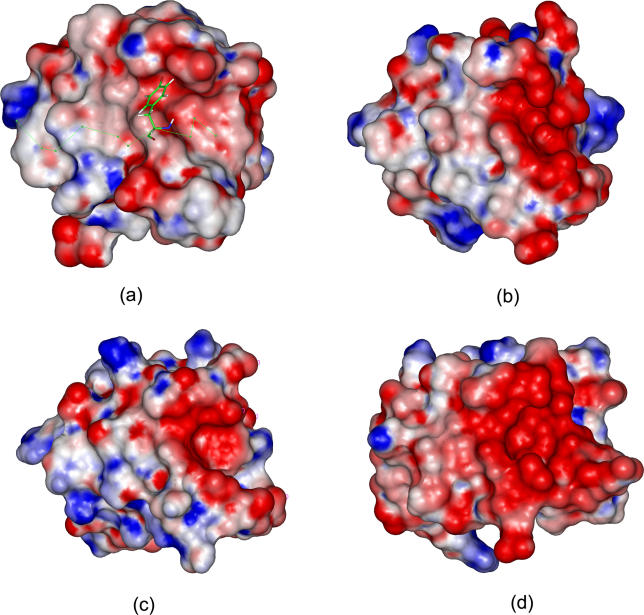
The Electrostatic Potentials of the Peptide-Binding Interfaces for Four SH3 Domains (A) 1bbz, (B) 1cka, (C) 1gbq, and (D) 1bb9. The scale of gradation was from −5 kT/e to +5 kT/e corresponding to red color to blue color. The electrostatic potentials of proteins were calculated using the Delphi module in Insight II. The salt concentration was set to 0.0 M because electrostatic potentials had small changes in the range of the experimental salt concentrations. The internal and external dielectric constants were set to 1 and 80, respectively. Electrostatic potentials were computed using a grid space of 0.5 Å with the focusing technique. The structures of the four SH3 domains were aligned using the Homology module in Insight II. The Tyr residue at P_−3_ in peptide APSYSPPPPP was shown in stick.

Our analysis showed that Trp, Phe, Tyr, Met, and Pro are favored at P_−3_ (Figure 3B), which is in good agreement with the study of Villanueva's et al. that the most favorable residues are Trp, Tyr, Phe, and Met (ordered based on the binding free energies) [22]. This observation is consistent with findings that an aromatic residue is favored at P_−3_ of mouse protein 3BP1, a known binder of the Abl SH3 domain [12, 23, 24]. The energy component analysis (Table S4) suggests that the strong preference of these four residues at this position is mainly due to the favorable non-polar contribution (ΔE_vdw_ + ΔG_SA_) upon peptide binding: Δ*E*_vdw_ + Δ*G*_SA_ for Trp, Tyr, Met, and Phe are −55.9, −55.4, −54.4, and −53.8 kcal/mol, respectively, which are stronger than the other 16 residues at this position. Interestingly, positive charged residues, Arg and Lys, are not favored at this position, which is in contrast to most SH3 domain-binding peptides. To investigate the reason, we have compared the electrostatic surfaces of four SH3 domains, Abl tyrosine kinase SH3 domain (PDB entry 1bbz) [25], c-Crk N-terminal SH3 domain (PDB entry 1cka) [26], Grb2 N-terminal SH3 domain (PDB entry 1gbq) [27], and rat amphiphysin-2 SH3 domain (PDB entry 1bb9) [28] (Figure 4). Electrostatic potentials were calculated by solving the Poisson-Boltzmann equation using the Delphi program [29] in Insight II [30]. We find that the rat amphiphysin-2 SH3 domain has the largest areas of negative electrostatic potentials, which is mainly due to the acidic residues in Arg-Thr and extended n-Src loops of the domain. The large patch of negative electrostatic potential explains why the amphiphysin SH3 domain specifically recognizes the PXRPXR motif with two positively charged Arg residues. The c-Crk N-terminal and the Grb2 N-terminal SH3 domain have distinct but relatively small negative potential near the Arg-Thr and n-Src loop, which may explain why these two SH3 domains bind to peptides only possessing a single positively charged residue. Compared with the other three SH3 domains, the Abl SH3 domain does not possess remarkable and continuous distribution of the negative electrostatic potentials and therefore positively charged residues are not strongly preferred.

Proline is highly preferred at positions P_0_ and P_3_. We find that Pro at P_0_ has favorable interactions with Phe9 and Tyr52 (−1.0 kcal/mol and −1.0 kcal/mol) (Table S7), as well as favorable van der Waals interaction with Pro49 (−0.8 kcal/mol). Experimental and theoretical studies have focused on understanding how SH3 domains recognize the core motif PXXP [31, 32]. Proline is the most favorable residue at this position based on our free energy calculation (Figure 3C). When only the non-polar contribution (Δ*E*_vdw_ + Δ*G*_SA_) is considered, the peptide with Pro has the strongest interaction with SH3 (−54.8 kcal/mol) (Table S5), which agrees with the study of Wang et al. [31]. In addition to the non-polar contribution, we found the less unfavorable desolvation-free energy is also an important factor for the preference of proline. For example, the residues at P_0_ in peptides 16, 19, and 20 (Table S5) all have relatively small side chains (Thr, Val, and Pro, respectively) and have similar protein-ligand electrostatic interactions Δ*E*_ele_. When the polar contributions of desolvation-free energies Δ*G*_PB_ were considered, the peptide 20 has the least unfavorable contribution (Δ*E*_ele_ + Δ*G*_PB_ = + 20.5 kcal/mol). We believe that upon peptide binding the desolvation cost for the nitrogen-substituted atom in Pro may be less than that of the non-substituted nitrogen atom in the other amino acids, because the non-substituted nitrogen atom can be easily polarized by the solvent [32].

Proline is also strongly selected at P_3_ in almost all SH3 domain-binding peptides. From the calculated binding free energies (Figure 3D), it is interesting to find that, although Pro is preferred at this position by the Abl SH3 domain, the preference is not very strong. Proline at this position can be mutated to several other residues including Trp, Phe, Met, His, Leu, Gln, and Tyr, and the mutated peptides still have relatively strong binding free energies. Our calculation seems to contrast with what has been suggested about the critical role of Pro at P_3_ in SH3 ligands [10]. However, in the mutation experiments reported by Pisabarro et al. [17], Pro at P_3_ in peptide APTYPPPLPP was mutated to His, Leu, and Tyr, and the binding affinities of the mutated peptides were only slightly decreased from −7.1 kcal/mol to −6.2, −6.2, and −6.2 kcal/mol, respectively. These three favorable residues reported by Pisabarro et al. were also relatively favored in our predictions.

### Identifying Physiological Interacting Partners of the Abl SH3 Domain

**Figure 5:**
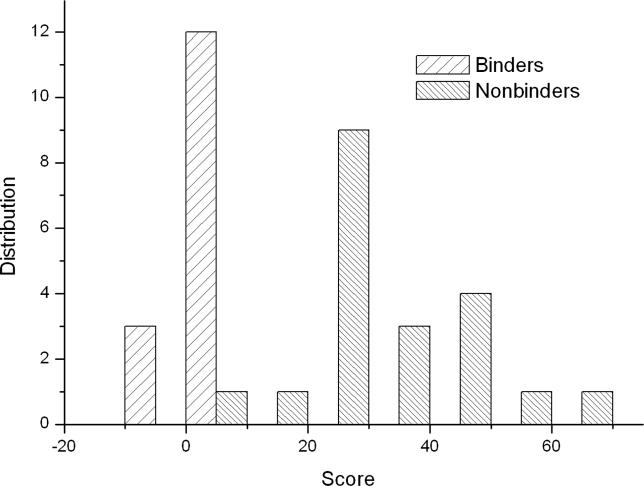
The Distribution of the Predicted Binding Free Energies Based on SPMFEP for Binders and Non-Binders

Based on the comparison between the experimental and calculated results, we have shown that the VM method can determine the binding motif of the Abl SH3 domain. The difference between the binding free energies of the mutated peptide at each position and the template peptide APSYSPPPPP, called SPMFEP, can be used as a position specific scoring matrix to predict the binding affinities of peptides (Table 2). To evaluate the performance of SPMFEP, the 15 binders and 20 non-binders were first scored using SPMFEP. Two obvious distributions for binders and non-binders can be observed (Figure 5), indicating that binders and non-binders can be successfully distinguished by SPMFEP. The binding affinity of peptide A4 was under-estimated. From our analysis (see above), we know that Phe at P_−6_ in the peptide A4 is favorable in the hydrophobic binding pocket that is originally occupied by Pro at P_−5_ in the template peptide. Consequently, the residues at P_−6_ and P_−5_ may interact with each other. In SPMFEP, the inter-dependence between positions is not considered. In fact, all methods using a position specific scoring matrix, such as Scansite [13], have the same limitations. Overall, SPMFEP performs well on the selected 35 peptides.

**Table 2:**
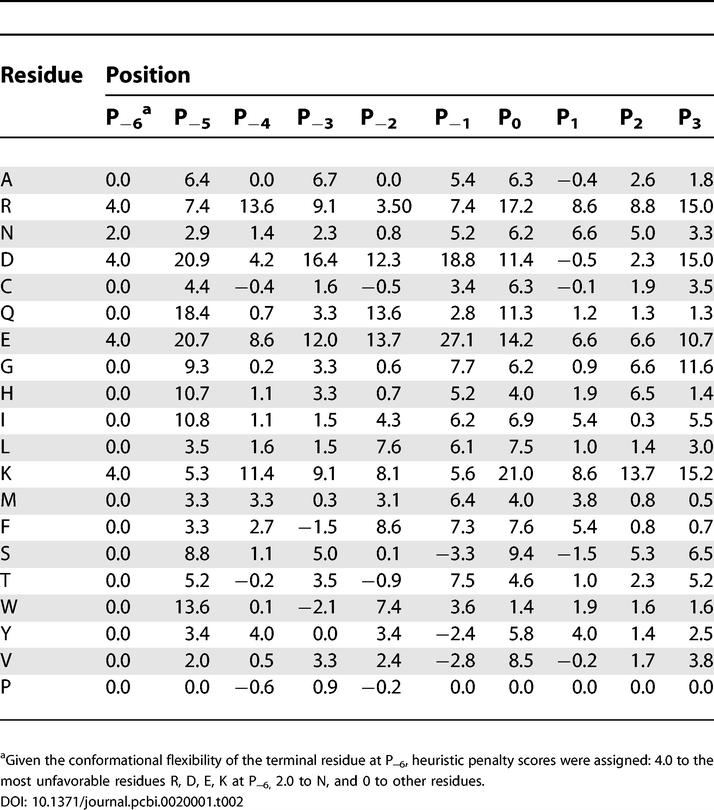
SPMFEPs of the Abl SH3 Domain

We next scanned the SWISS-PROT using SPMFEP to predict interacting partners of the Abl SH3 domain. There are about 6.2 × 10^7^ ten-residue-long peptides in the current SWISS-PROT database (May 2005), in which about 218,540 ten-residue-long peptides have the PXXP motif. Only about 2,600 peptides have scores smaller than two, which are in the top 0.005% (the top 600 peptide sequences in 353 unique human proteins are listed in Table S9). We first carefully examined the top ten candidates in the human proteome (Table 3), among which WASF1 and EVL are known interacting partners of the Abl SH3 domain [33, 34]. WASF4 is a homology of WASF1 and is in the same protein family. SEM6A is a homology of the mouse protein SEM6D, a known Abl SH3 domain-binding protein, and is therefore likely to be a true binder [35]. In total, we have identified two known binders and two candidates highly supported by experimental evidence, among the top ten peptides, which is a surprisingly good result. As a comparison, the top ten human peptides in the Scansite search are [13] 3BP2, RX, RBMG, TACT, PRL3, SCA3, AT19, AD08, DYN2, and SEP4, among which only 3BP2, the homology of a known binder (mouse protein 3BP2), is likely to be a true binder [36] but no binding information is found for all other candidates in BIND [36] and MINT [37] databases to interact with Abl protein or the Abl SH3 domain. If only considering the top ten candidates, the SPMFEP method based on VM performs better than Scansite on identifying the interacting partners of the Abl SH3 domain.

**Table 3:**
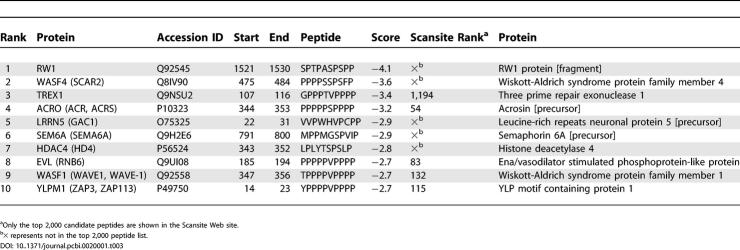
Top Ten Binding Partner Candidates of the Abl SH3 Domain Identified by SPMFEP

In MINT [37] and BIND [36], 44 non-redundant proteins have been identified to directly interact with the protein Abl, and 13 of them, including five mouse proteins and eight human proteins, bind to the Abl SH3 domain. We compared the performance of SPMFEP, Scansite [13], and iSPOT [14], to identify these 13 proteins (Table 4). The top 600 candidates found in human proteins and the top 2,000 candidates found in all proteins were saved for further analyses.

**Table 4:**
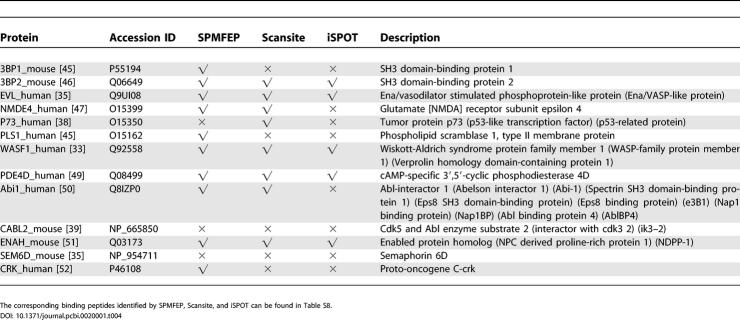
Comparison of Predictions of SPMFEP, Scansite, and iSPOT on 13 Known Interacting Proteins of the Abl SH3 Domain

SPMFEP can successfully identify ten known binders: seven of the eight human proteins and three of the five mouse proteins (Table 4 and Table S8). 3BP1_mouse and 3BP2_mouse are not ranked highly, considering all proteins (1,393 and 1,895) but they are in the top 500 if only mouse proteins are considered. The human homologies of 3BP1_mouse and 3BP2_mouse are in the top 600 candidates (249 for 3BP1_human and 502 for 3BP2_human) (Table S9) and it is reasonable to believe that they are true binders of the Abl SH3 domain. Overall, we have successfully identified most of the known binders of the Abl SH3 domain.

Scansite can identify eight known binders of the Abl SH3 domain (Table 4). P73_human, not identified by SPMFEP, is ranked 321 in the Scansite result. Agami et al. [38] reported that a P73 mutant P338A could not form stable P73-Abl complexes. If P73 interacts with Abl by ten-residue-long peptide segment, this peptide segment should be AFKQSPPAVP, which is the same as the peptide identified by Scansite. Based on our VM analysis and mutation experiments by Pisabarro and Serrano [17], Phe at P_−5_ and Lys at P_−4_ are not favored. Because Scansite considers 15-residue-long peptides rather than ten-residue-long peptides in SPMFEP, it is likely that the five additional residues may contribute favorably to binding. It is not surprising that the longer the peptide, the more specific, but less sensitive, are the predictions.

Using iSPOT, we can only correctly identify five binders (Table 4). In iSPOT, the scoring matrix was derived from position-specific contacts based on six SH3-peptide or SH3-protein complex structures [14]. The accuracy of the matrix is limited by the relatively small number of residue-resident contacts found between SH3 domains and their binding peptides to fill the 27 × 10 × 10 × 10 position-specific contact matrix.

CABL2_human and SEM6D_mouse (Table 4) cannot be identified by all the three methods. Experiments [35, 39] have shown that their interactions with Abl are mediated by the interaction between the proline-rich region and the Abl SH3 domain. Since the scoring matrix used by all the three methods does not consider dependence between positions, we suspect that synergistic interactions may exist between positions within or beyond the proline-rich regions of the two proteins.

## Discussion

In this study, we have demonstrated that the MM/PBSA method can accurately calculate the binding free energies between the Abl SH3 domain and its peptide ligands. Examination of each component of the binding free energy shows that, besides the non-bonded interactions and desolvation effect, the change of the conformational energies of the peptides upon binding is also crucial to determine the binding specificity of the domain. These results are encouraging to apply MD simulation and free energy calculation to understand the molecular mechanism of other domain-peptide and protein-protein interactions.

We have also shown that the VM method can precisely determine the sequence motif recognized by the Abl SH3 domain. The experimental scheme of the VM method is totally different from those of the current peptide library experiments in the following ways: 1) produce all possible peptides that are one amino acid different from the template peptide; (2) measure the “binding” affinities of all these peptides; and, (3) generate a scoring matrix based on the binding affinity differences between the mutated and the template peptides to determine the binding motif.

There are advantages of this scheme. First, the preference of an amino acid is quantitatively measured, based on the binding affinity of the peptide, which at least partially overcomes the sampling difficulty in the current peptide library experiment. Ideally, to determine the binding motif of a domain, one should examine the binding between the domain and all possible peptides of a given length, and align all binding peptides to calculate the frequency of each amino acid occurring at each position. In reality, there are usually only 10^7^–10^10^ peptides in the library, due to the limit of time and cost. If the length of the binding peptide is ten, there are 10^10^–10^13^ possible peptides and the coverage of the peptide sequence space by the peptide library is about 10^−6^–10^−3^. If 15-residue-long peptides are considered as in Scansite, the coverage drops dramatically to 10^−12^–10^−9^. By measuring the relative preference of every amino acid based on the peptide-binding affinities, one overcomes this insufficient sampling issue and mimics the ideal procedure given the position-independence assumption. Second, the VM method can evaluate the penalties of unfavorable amino acids even the peptide do not really bind to the protein, which is very hard if not impossible in the experimental approaches. Third, since the scoring matrix is obtained by taking the difference between the template and the mutated peptides that are only one amino acid different from the template peptide, some errors due to insufficient sampling of conformational space and/or inaccurate free energy calculation can be cancelled.

There are two major hurdles of applying the VM method in a high-throughput manner. First, the MD simulation and free energy calculations are time-consuming. Second, a domain-peptide complex structure is required. Given the fast pace of advancement of computer power and structural genomics/homology modeling, we believe that the VM method will become more and more useful.

## Materials and Methods

### MD simulations.

MD simulations were performed on the 15 binders and 20 non-binders of the Abl SH3 domain using the AMBER 8 simulation package [19] and AMBER03 force field [40]. The amino acid sequences and the experimentally determined binding affinities of the 15 binders are shown in Table 1 [17]. Ten peptides, B1 to B10, were randomly selected from the human proteome and are considered as non-binders (Table S1). Ten peptides, C1 to C10, do not bind to the Abl SH3 domain but are Class I binders of other SH3 domains [12]: C1 and C2 bind to the Src SH3 domains, C3 and C4 bind to the Yes SH3 domain, and C5 to C10 bind to the Grb2 N-terminal SH3 domain (Table S1). We chose the crystal structure of the peptide APSYSPPPPP complexed with the Abl SH3 domain (Class I binder and the PDB entry is 1bbz) [25] as the template and mutated it to other peptides using the *scap* program [41]. The complex was solvated in a rectangular box of about 3,000 TIP3P water molecules so that the boundary of the box is at least 9 Å away from any solute atom. Counter-ions of Na^+^ were placed based on the Columbic potential to keep the whole system neutral. Particle Mesh Ewald (PME) was employed to consider the long-range electrostatic interactions [42]. Following 2,000 steps of minimization, a 1.2 ns (30 ps temperature increase from 10 °K to 300 °K and 1.17 ns equilibration and data collection) MD simulation with a 2.0 fs time step was performed on each complex. The SHAKE procedure was employed to constrain hydrogen atoms [43] during MD and all heavy atoms of SH3 were restrained using a 5 kcal·mol^−1^·Å^−2^ harmonic force (see discussion in Supporting Information).

To determine the conformational energy of unbound peptide in solvent, 2.0 ns MD simulation was conducted on each peptide. Each peptide was solvated in a water box of about 1,600 water molecules, which extended 10 Å away from any peptide atom. 1,000 steps of minimization were followed by a 2.0 ns MD simulation for equilibration and data collection using the same set-up as described above.

### Free energy calculations using the MM/PBSA method.

The binding free energy is calculated as:


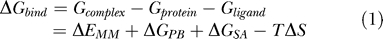


where Δ*E_MM_* is the molecular mechanics interaction energy between the SH3 domain and the peptide, Δ*G_PB_* and Δ*G_SA_* are the electrostatic and non-polar contributions to desolvation upon peptide binding, respectively, and −*T*Δ*S* is the conformational entropy change. To consider the conformational flexibility of the peptide, we ran two separate MD simulations on the complex and the free peptide to calculate the binding free energy [16].

Δ*E_MM_* was calculated using the *sander* program in AMBER 8 [19]. Δ*G_PB_* was calculated using the *pbsa* program in AMBER 8. The grid size used to solve the Poisson-Boltzmann equation was 0.5 Å, and the values of interior dielectric constant and exterior dielectric constant were set to 1 and 80, respectively (the influence of the interior dielectric constant value to the free energy calculation is discussed in Supporting Information). Δ*G_SA_* was estimated from the surface area [16, 44]. The peptide-SH3 interaction energies were calculated from 150 snapshots taken from 300 ps to 1.2 ns MD simulation trajectories of the complex. 160 snapshots taken from 400 ps to 2.0 ns MD simulations on the unbound peptides were used to calculate the conformational energy change for the peptides.

The normal mode analysis was performed to estimate the vibrational component of the entropy using the *nmode* program in AMBER 8 [19]. In the absence of solvent, the structures (complex, SH3, and peptide) were minimized with no cutoff for non-bonded interactions, by using conjugate gradient and then Newton-Raphson minimizations until the root mean square of the elements of gradient vector was less than 5 × 10^−5^ kcal/mol. Then, normal mode calculations were carried out with no cutoff for non-bonded interactions. A distance-dependent dielectric constant (ɛ = 4*R*_ij_) was used to mimic solvent screening. Frequencies of the vibrational modes were computed at 300K for these minimized structures and using a harmonic approximation of the energies. Due to the high computational demand, only 25 snapshots taken from MD were used to estimate −TΔS.

### The VM method.

To investigate the preference of residues at each position, systematic single point mutation was performed on the peptide. The peptide APSYSPPPPP in the crystal structure 1bbz was used as the template. Each residue of the peptide was mutated to the other 19 residues using the *scap* program [41]. Minimization, MD simulations, and MM/PBSA calculations were performed on all 190 mutated complexes, as well as on the free peptides using the same set-up described above. Assuming mutating a single residue of the peptide did not significantly change the peptide conformation, we did not include the conformational entropy in the comparison of 20 residues at each position.

### Single point mutation free energy profile and database scan.

The SPMFEP is a 10 × 20 matrix, which represents the difference between the binding free energies of the mutated peptides and the template peptide APSYSPPPPP (Table 2). SPMFEP can be used as a position specific scoring matrix. The score of each peptide is calculated as: 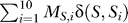 , where *M_S,i_* is the score of the amino acid *S* at *i^th^* position in the SPMFEP and *S_i_* is the amino acid at the *i^th^* position of the peptide. All ten-residue-long peptides in the SWISS-PROT database (release 46.4) were scored using the SPMFEP. The Perl script used for the database scan is available upon request.

## Supporting Information

Figure S1 The Correlations between the Experimental and Calculated Relative Binding Free Energies Using an Interior Dielectric Constant of 2 (249 KB TIF)

Figure S2 The Superposition of 23 Snapshots Extracted from the MD Trajectory from 0.1 ns to 1.2 ns The structure of SH3 shown here was extracted from the snapshot at 0.1 ns. The residues at P_3_, P_−3_, P_−4_, P_−5,_ and P_−6_ of the peptide are colored in red and other residues are colored according to residue type defined in Insight II.
(2.9 MB TIF)

Figure S3 The Fluctuations of the Free Energy Components PB represents the polar contribution to the solvation free energy (ΔGPB). Electrostatic means the electrostatic interaction between the peptide and the SH3 domain (ΔEele), and van de Waals means the van de Waals interactions between the peptide and the SH3 domain (ΔEvdw).
(358 KB TIF)

Figure S4 The Interactions between Peptide FGTYPPPLPP and the Abl SH3 Domain Two residues, Trp36 and Trp47, are shown in stick, and the peptide is shown in ball-and-stick.
(942 KB TIF)

Protocol S1 The MM/PBSA Calculations Protocols and the Conformational Changes of Ligands in Peptide Binding (42 KB DOC)

Table S1 The Energetic Components and Binding Affinities for 20 Non-Binders (kcal/mol) (66 KB DOC)

Table S2 The Energetic Components and Binding Affinities for the 15 Peptide Ligands of the Abl SH3 Domain Using Interior Dielectric Constant of 2 (kcal/mol) (56 KB DOC)

Table S3 The Binding Free Energies for 20 Peptides Mutated at Position P_−5_ (kcal/mol) (63 KB DOC)

Table S4 The Binding Free Energies for 20 Peptides Mutated at Position P_−3_ (kcal/mol) (62 KB DOC)

Table S5 The Binding Free Energies for 20 Peptides Mutated at Position P_0_ (kcal/mol) (61 KB DOC)

Table S6 The Binding Free Energies for 20 Peptides Mutated at Position P_3_ (kcal/mol) (65 KB DOC)

Table S7 The Contribution of Each Residue in Peptide APSYSPPPPP to the SH3 Binding (kcal/mol) (57 KB DOC)

Table S8 The High-Rank Peptides Selected by PMFEP, Scansite, and iSPOT in 13 Abl SH3-Binding Proteins (54 KB DOC)

Table S9 The Top 600 Sequences Found in Human Proteins from Database Search (643 KB DOC)

### Accession Numbers

The Swiss-Prot and TrEMBL (http://www.expasy.org/sprot) accession numbers for the genes and gene products discussed in this paper are: 1bb9 (1bb9), 1bbz (CAB04591), 1cka (CAA18266), 1gbq (1gbqa), 3BP1_human (Q9Y3L3), 3BP1_mouse (P55194), 3BP2 (P78314), 3BP2_mouse (Q06649), AD08 (P78325), AT19 (Q8TE59), CABL2_human (NP_665850), DYN2 (P50570), EVL (Q9UI08), P73_human (O15350), PRL3 (P02814), RBMG (Q9UPN6), RX (Q9Y2V3), SCA3 (O14828), SEM6A (Q9H2E6), SEM6D (Q76KF3), SEM6D_mouse (NP_954711), SEP4 (O43236), TACT (P40200), WASF1 (Q92558), and WASF4 (Q8IV90).

## Abbreviations

MD: molecular dynamics
MM/PBSA: Molecular Mechanics/Poisson-Boltzmann Solvent Area
SD: standard deviation
SH2: Src Homology 2 domain
SH3: Src Homology 3 domain
SPMFEP: single position mutation free energy profile
VM: virtual mutagenesis
