# Supplementary figures and images for: Computational Analysis and Prediction of the Binding Motif and Protein Interacting Partners of the Abl SH3 Domain
Source: PLoS Comput Biol. 2006 Jan 27;2(1):e1. doi: 10.1371/journal.pcbi.0020001 (PMC1356089; doi:10.1371/journal.pcbi.0020001)

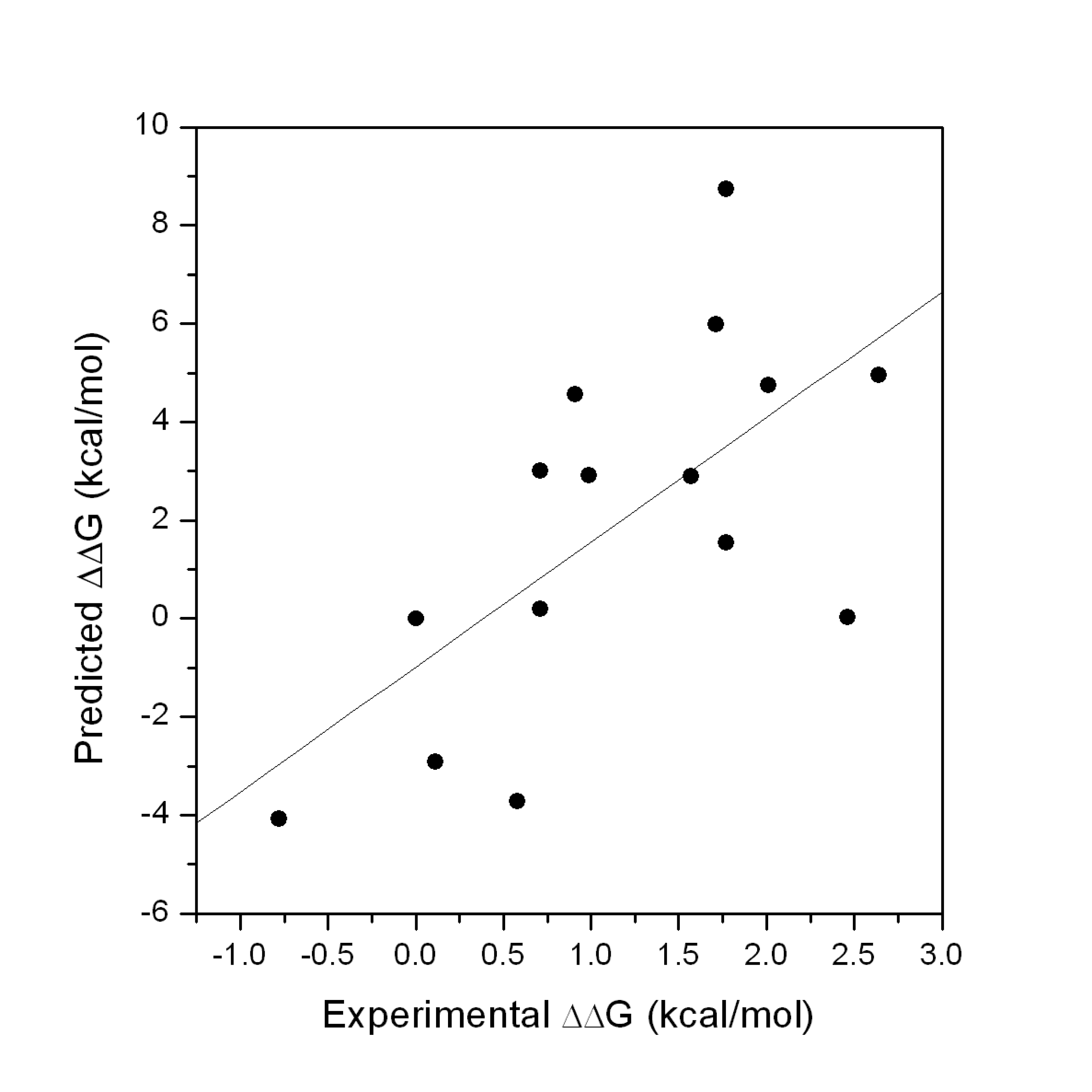

Supplement: Figure S1 — (249 KB TIF) [file pcbi.0020001.sg001.tif]

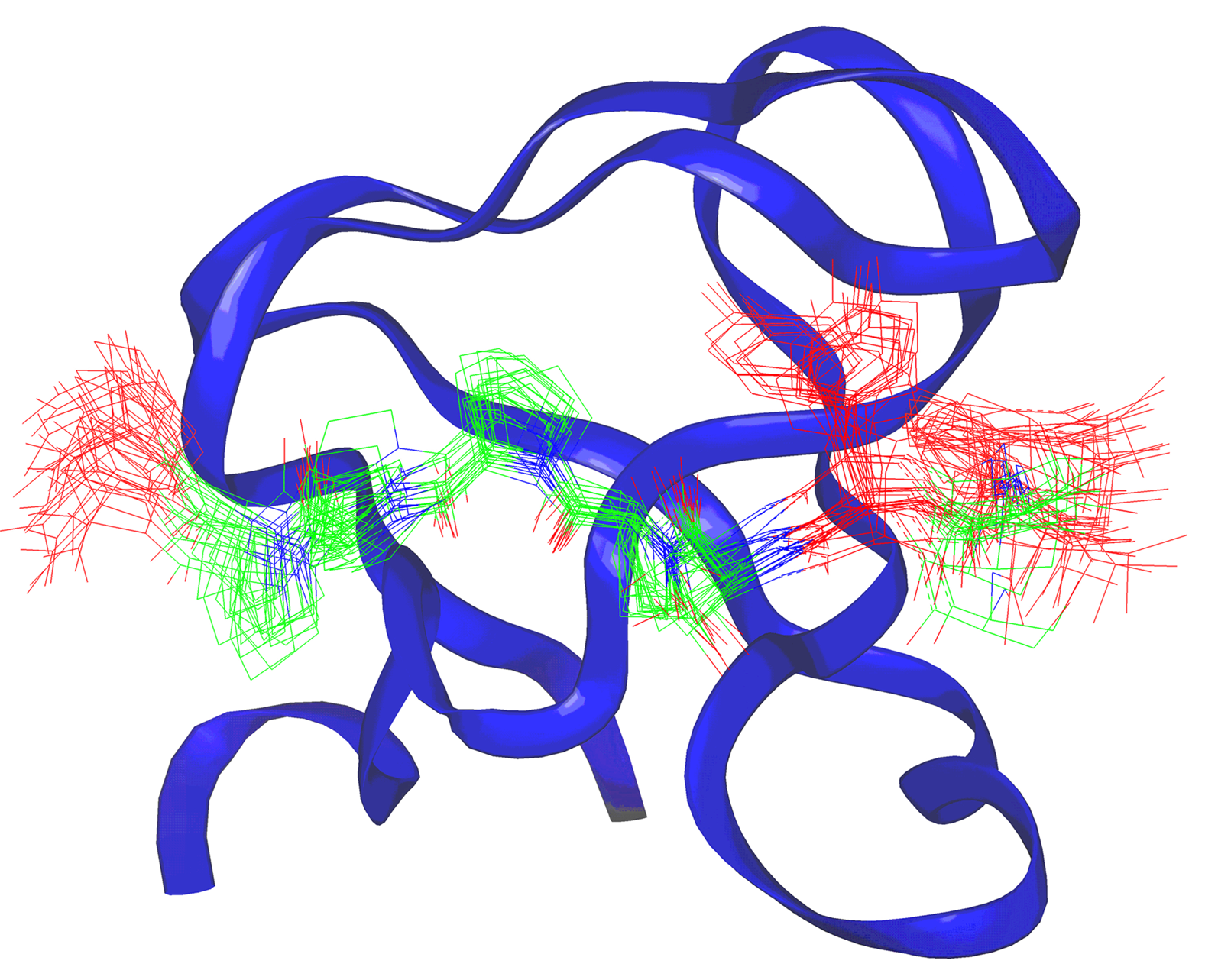

Supplement: Figure S2 — The structure of SH3 shown here was extracted from the snapshot at 0.1 ns. The residues at P3, P−3, P−4, P−5, and P−6 of the peptide are colored in red and other residues are colored according to residue type defined in Insight II. (2.9 MB TIF) [file pcbi.0020001.sg002.tif]

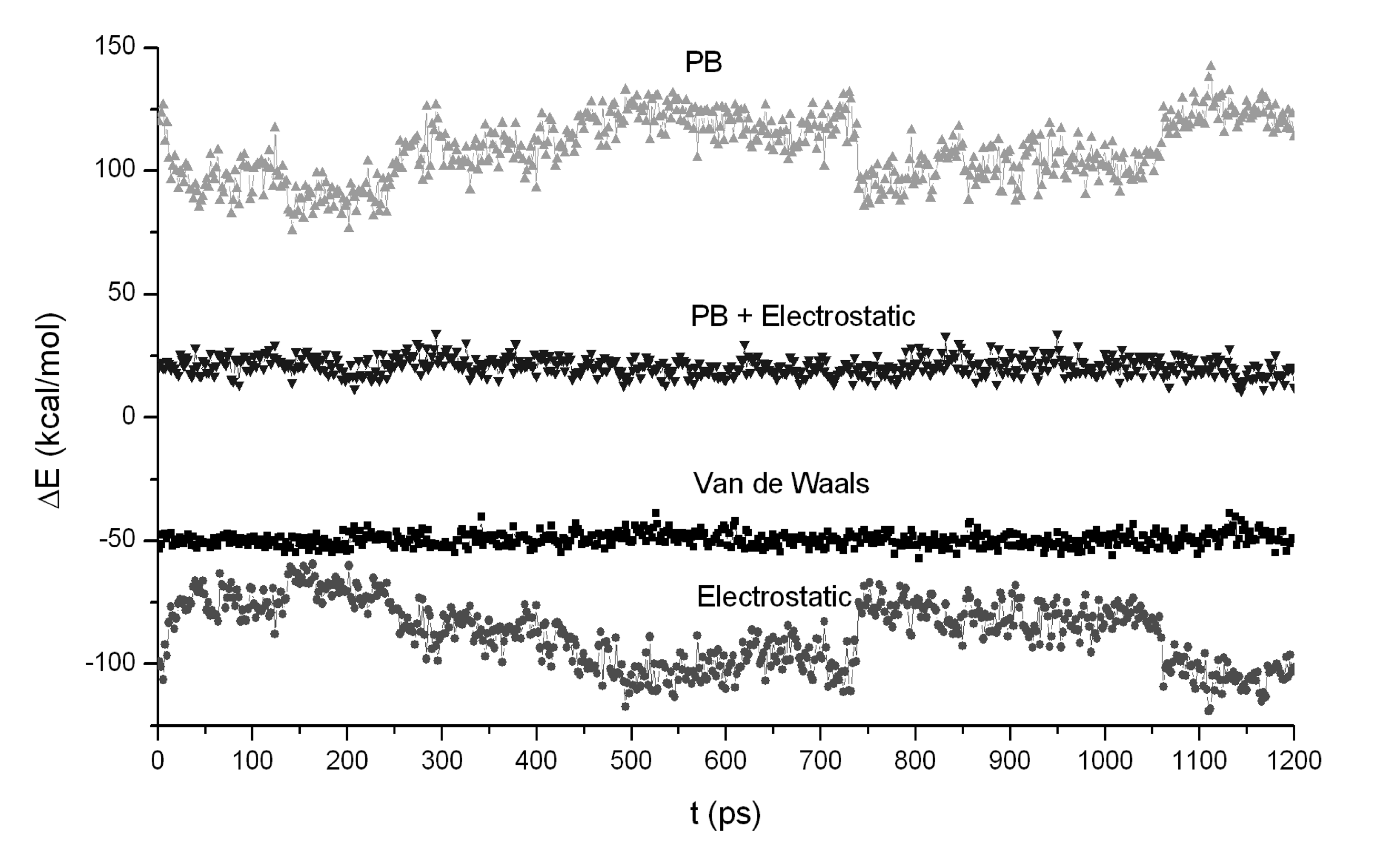

Supplement: Figure S3 — PB represents the polar contribution to the solvation free energy (ΔGPB). Electrostatic means the electrostatic interaction between the peptide and the SH3 domain (ΔEele), and van de Waals means the van de Waals interactions between the peptide and the SH3 domain (ΔEvdw). (358 KB TIF) [file pcbi.0020001.sg003.tif]

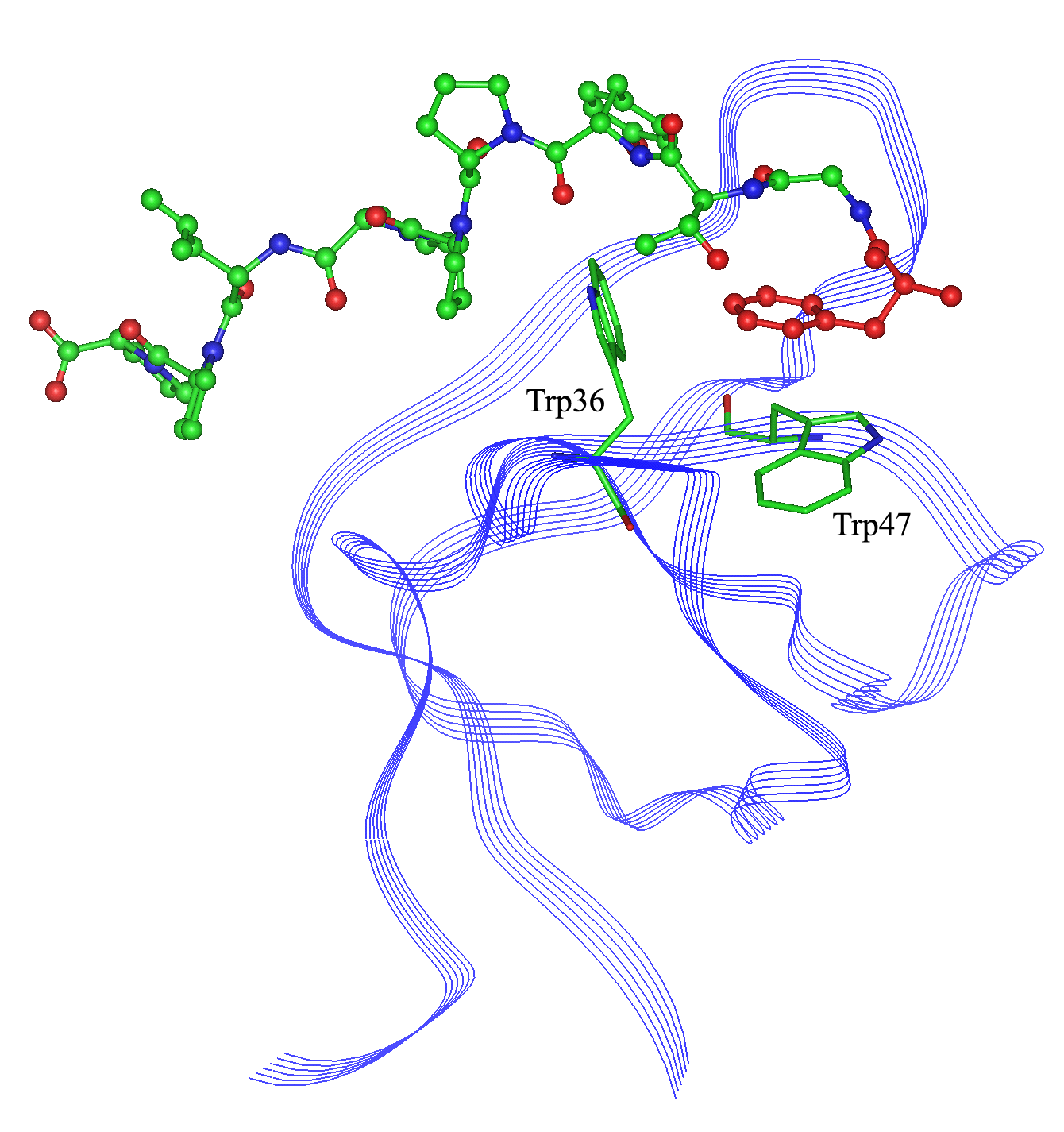

Supplement: Figure S4 — Two residues, Trp36 and Trp47, are shown in stick, and the peptide is shown in ball-and-stick. (942 KB TIF) [file pcbi.0020001.sg004.tif]
