# Supplementary material for: Computational Analysis and Prediction of the Binding Motif and Protein Interacting Partners of the Abl SH3 Domain
Source: PLoS Comput Biol. 2006 Jan 27;2(1):e1. doi: 10.1371/journal.pcbi.0020001 (PMC1356089; doi:10.1371/journal.pcbi.0020001)
Supplement: Protocol S1 — (42 KB DOC) [file pcbi.0020001.sd001.doc]

**Part1. MM/PBSA calculations**

In MM/PBSA calculations, the affinity of a peptide binding to a protein can be estimated according to equation 1 in the text by two procedures: (a) run separate trajectories of complex, protein, and ligand or (b) evaluate all three terms in equation 1 using just the snapshots from a trajectory on the complex. Certainly, procedure (b) is much faster than procedure (a). Some applications have shown that procedure (b) is a good approximation to procedure (a) in most applications [1]. For protein/peptide or protein/protein system, the change of the conformational energy of protein and peptide should be considered better by using separate MD simulations. For the systems studied here, the conformations of some peptides may be significantly different in bound and unbound states. Moreover, for different peptides, the change of conformational energy may not be treated as a constant due to the flexibility of peptide in the binding pocket. The binding free energies should be calculated as the following:

$\begin{matrix} \Delta G_{bind}=G_{complex}-G_{protein}-G_{ligand} \\ =\Delta E_{MM}+\Delta G_{PB}+\Delta G_{NP}-T\Delta S \\ =\Delta E_{ele}+\Delta E_{vdw}+\Delta E_{conf\_pep}+\Delta E_{conf\_SH3}+\Delta G_{PB}+\Delta G_{SA}-T\Delta S \end{matrix}$ (1)

$$\Delta G_{conf\_pep}=G_{lig\_bound}-G_{lig\_unbound}$$

$$\Delta G_{conf\_SH3}=G_{SH3\_bound}-G_{SH3\_unbound}$$

Where Δ*E_ele_ and* Δ*E_vdw_* are the electrostatic and the van der Waals interactions between the peptide and the SH3 domain, respectively; Δ*G_PB_* and Δ*G_SA_* are the polar and the nonpolar contributions of the solvation free energies upon peptide binding, respectively; Δ*Gconf_pep* and Δ*Gconf_SH3* are the differences between the conformational energy of the peptide and the Abl SH3 domain in bound and unbound states, respectively; *Glig_bound* and *Glig_unboud* are the conformational energies of the peptide in bound and unbound states, respectively; *GSH3_bound* and *GSH3_unboud* are the conformational energies of the SH3 domain in bound and unbound states, respectively.

In our calculation, Δ*Gconf_SH3* was not included and the reasons are the following. The SH3 domain is a small protein (50-70 amino acids) and relatively flexible compared with other proteins such as HIV protease (about 200 amino acids). The value of conformational energy of the SH3 domain is several orders of magnitude larger than those of peptides and the interacting energies. To avoid the error of taking difference between two large values, Δ*Gconf_SH3* was not considered [2]. In fact, the conformation of the Abl SH3 domain was restrained to the crystal structure using a 5 kcal·mol^-1^·Å^-2^ harmonic force in the MD simulations to avoid the significant conformational deviation caused by insufficient sampling and inaccuracy of the force field.

**Part2. The influence of interior dielectric constant to the predicted peptide binding**

The absolute values of the calculated binding free energies can be influenced by the value of the interior dielectric constant. In the current studies, a value of 1 was used for the interior dielectric constant of protein and peptides. In some other applications [3-4], a dielectric constant of 2 or 4 was used. When the interior dielectric constant of 1 was replaced by *n*, the binding free energy shown in Equation 1 was transformed to the following equation:

$\begin{matrix} \Delta G_{bind}=G_{complex}-G_{protein}-G_{ligand} \\ =\Delta E_{MM}+\Delta G_{PB}+\Delta G_{NP}-T\Delta S \\ =\frac{1}{n}\Delta E_{ele}+\Delta E_{vdw}+\Delta E_{conf\_pep}+\Delta G_{PB}^{n-80}+\Delta G_{SA}-T\Delta S \end{matrix}$ (2)

$$\Delta E_{conf\_pep}=\frac{1}{n}\Delta E_{ele\_conf\_pep}+\Delta E_{vdw\_conf\_pep}+\Delta E_{PB\_conf\_pep}+\Delta E_{SA\_conf\_pep}$$

where the *n* is the interior dielectric constant. The binding free energies and the contributions of all energy terms using a dielectric constant of 2 are listed in Table S2. The correlation between the experimental binding free energies and the predicted values is shown in Figure S1. A linear correlation coefficient *r*=0.66 can be observed, which is smaller than that using the interior dielectric constant of 1. We noticed that the absolute values of the binding free energies are more negative using interior dielectric constant 2 than using dielectric constant 1. It is not surprising because the positive value of Δ*E*_ele_+Δ*G*_PB_ was nearly scaled by the interior dielectric constant in the system we studied. The selection of suitable interior dielectric constant is crucial. However, as shown in many studies, it is difficult to define a unique dielectric constant for various protein-ligand complexes [3-5]. The optimal value for one protein family is not necessarily transferable to another protein family because the interior dielectric constant depends on the polarization state of each system.

Accurately calculating absolute binding free energy between peptide and protein is a very challenging task. Many efforts have been made to tackle this problem. For example, in 1997, Froloff et al. have applied MD simulations and continuum solvation model to calculate the binding free energy for several protein/peptide systems [6]. Since we are interested in the binding specificity of the Abl SH3 domain, relative binding free energies between peptides are more important than the absolute binding free energy values in this study. Therefore, we used value 1 for the interior dielectric constant.

**Part3. Conformational flexibility of the peptide in the complex**.

The MD trajectories showed that the peptides were quite flexible even in the complex (Figure S2), particularly the N- and C-terminal residues that were relatively less restrained by the Abl SH3 domain than the core residues at positions P_-2_ to P_2_. In addition, the side chain of Tyr at P_-3_ was also very flexible. Therefore, long MD simulations were necessary to sample the conformational flexibility of the peptide. Further examination of the fluctuation of the individual components to binding free energies reveals that the electrostatic interactions between the SH3 domain and peptide (Δ*E*_ele_) and the electrostatic contribution of desolvation upon peptide binding (Δ*G*_PB_) fluctuate much more than the van der Waals interactions while Δ*E*_ele_+Δ*G*_PB_ is quite stable because the two terms are cancelled out (Figure S3).

**References**

1. Kollman PA, Massova I, Reyes C, Kuhn B, Huo SH, et al. (2000) Calculating structures and free energies of complex molecules: combining molecular mechanics and continuum models. Acc. Chem. Res. 33: 889-897.
2. Gohlke H, Case DA (2004) Converging free energy estimates: MM-PB(GB)SA studies on the protein-protein complex Ras-Raf  J. Comput. Chem. 25: 238-250.
3. Wang W, Lim WA, Jakalian A, Wang J, Wang JM, et al. (2001) An analysis of the interactions between the Sem-5 SH3 domain and its ligands using molecular dynamics, free energy calculations, and sequence analysis. J. Am. Chem. Soc. 123: 3986-3994.
4. Wang W, Kollman PA (2000) Free energy calculations on dimer stability of the HIV protease using molecular dynamics and a continuum solvent model. J. Mol. Biol. 303: 567-582.
5. Schapira M, Totrov M, Abagyan R (1999) Prediction of the binding energy for small molecules, peptides and proteins. J. Mol. Recognit. 12: 177-190.
6. Froloff N, Windemuth A, Honig B (1997) On the calculation of binding free energies using continuum methods: Application to MHC class I protein-peptide interactions. Protein Sci. 6: 1293-1301.
7. Cicchetti P, Mayer BJ, Thiel G, Baltimore D (1992) identification of a protein that binds to the SH3 region of abi and is similar to bcr and gap-rho. Science 257: 803-806.
8. Ren R, Mayer BJ, Cicchetti P, Baltimore D (1993) Identification of a 10-amino acid proline-rich SH3 binding-site. Science 259, 1157-1161.
9. Lambrechts A, Kwiatkowski AV, Lanier LM, Bear JE, Vandekerckhove J, et al. (2000) cAMP-dependent protein kinase phosphorylation of EVL, a mena/VASP relative, regulates its interaction with actin and SH3 domains. J. Biol. Chem. 275: 36143-36151.
10. Glover RT, Angiolieri M, Kelly S, Monaghan DT, Wang JY, et al. (2000) Interaction of the N-methyl-D-aspartic acid receptor NR2D subunit with the c-Abl tyrosine kinase. J. Biol. Chem. 275: 12725-12729.
11. Agami R, Blandino G, Oren M, Shaul Y (1999) Interaction of c-Abl and p73 alpha and their collaboration to induce apoptosis. Nature 399: 809-813.
12. Sun J, Zhao J, Schwartz MA, Wang JY, Wiedmer T, et al. (2001) c-Abl tyrosine kinase binds and phosphorylates phospholipid scramblase 1. J. Biol. Chem. 276: 28984-18990.
13. Westphal RS, Soderling SH, Alto NM, Langeberg LK, Scott JD (2000) Scar/WAVE-1, a Wiskott-Aldrich syndrome protein, assembles an actin-associated multi-kinase scaffold. EMBO J. 19: 4589-4600.
14. Beard MB, O'Connell JC, Bolger GB, Houslay MD (1999) The unique N-terminal domain of the cAMP phosphodiesterase PDE4D4 allows for interaction with specific SH3 domains. FEBS Lett. 22: 173-177
15. Yamamoto A, Suzuki T, Sakaki Y (2001) Isolation of hNap1BP which interacts with human Nap1 (NCKAP1) whose expression is down-regulated in Alzheimer's disease. Gene 271: 159-169.
16. Sato H, Nishimoto I, Matsuoka M (2002) ik3-2, a relative to ik3-1/Cables, is associated with cdk3, cdk5, and c-abl. Biochim. Biophys. Acta. 19: 157-163.
17. Gertler FB, Niebuhr K, Reinhard M, Wehland J, Soriano P (1996) Mena, a relative of VASP and Drosophila enabled, is implicated in the control of microfilament dynamics. Cell 87: 227-239.
18. Toyofuku T, Zhang H, Kumanogoh A, Takegahara N, Yabuki M, et al. (2004) Guidance of myocardial patterning in cardiac development by Sema6D reverse signalling. Nat. Cell Biol. 6: 1204-1211
19. Donaldson LW, Gish G, Pawson T, Kay LE, Forman-Kay JD (2002) Structure of a regulatory complex involving the Abl SH3 domain, the Crk SH2 domain, and a Crk-derived phosphopeptide. Proc. Natl. Acad. Sci. USA. 99, 14053-14058.
