# Supplementary material for: Computational Analysis and Prediction of the Binding Motif and Protein Interacting Partners of the Abl SH3 Domain
Source: PLoS Comput Biol. 2006 Jan 27;2(1):e1. doi: 10.1371/journal.pcbi.0020001 (PMC1356089; doi:10.1371/journal.pcbi.0020001)
Supplement: Table S1 — (66 KB DOC) [file pcbi.0020001.st001.doc]

Table S1. The energetic components and binding affinities for the 20 non-binders (kcal/mol)

| No. | Sequence | Δ*E*_ele_ | Δ*E*_vdw_ | Δ*G*_SA_ | Δ*G*_PB_ | TΔS | Glig_bound | Glig_unbound | ΔG_pred_ | ΔΔG_pred_ |
| --- | --- | --- | --- | --- | --- | --- | --- | --- | --- | --- |
| B1 | SKKEMQPTHP | -176.2  ±0.8 | -46.0  ±0.1 | -5.6  ±0.0 | 207.5  ±0.7 | -36.6  ±0.3 | -288.4  ±2.8 | -289.2  ±1.3 | 17.0  ±0.1 | 19.6 |
| B2 | ASQKMEPRAP | -173.0  ±7.2 | -35.5  ±1.2 | -4.9  ±0.1 | 214.3  ±8.2 | -35.5  ±0.3 | -357.1  ±0.3 | -361.5  ±0.3 | 40.8  ±1.2 | 43.3 |
| B3 | WELSSQPTIP | -17.9  ±0.5 | -55.8  ±0.1 | -6.1  ±0.0 | 58.8  ±1.6 | -43.1  ±0.1 | -258.5  ±1.4 | -260.0  ±0.5 | 23.7  ±1.1 | 26.3 |
| B4 | LAPASTPTSP | -102.7  ±14.6 | -39.8  ±1.1 | -5.1  ±0.1 | 121.5  ±12.4 | -33.0  ±0.5 | -134.3  ±1.9 | -138.3  ±1.1 | 11.0  ±1.4 | 13.6 |
| B5 | ASTPTSPSSP | -101.8  ±10.0 | -41.8  ±3.7 | -5.1  ±0.2 | 120.2  ±11.3 | -35.0  ±0.4 | -140.8  ±0.7 | -143.2  ±0.3 | 8.8  ±2.4 | 11.4 |
| B6 | SSPGLSPVPP | -65.6  ±2.0 | -37.3  ±1.6 | -4.3  ±0.2 | 84.6  ±4.5 | -33.3  ±0.6 | -66.3  ±2.0 | -66.9  ±1.8 | 11.3  ±0.8 | 13.8 |
| B7 | RGVLIEPVYP | -54.6  ±3.7 | -48.9  ±0.3 | -5.6  ±0.2 | 101.6  ±3.9 | -33.0  ±0.4 | -313.0  ±0.4 | -323.7  ±0.4 | 36.3  ±1.7 | 38.9 |
| B8 | DEPNLEPSWP | 101.7  ±6.8 | -48.3  ±0.7 | -5.5  ±0.1 | -46.8  ±5.7 | -34.0  ±0.3 | -302.6  ±1.5 | -291.3  ±0.4 | 23.8  ±2.0 | 26.4 |
| B9 | RLVGARPLLP | -208.6  ±2.7 | -51.0  ±2.2 | -5.6  ±0.1 | 245.5  ±1.8 | -35.5  ±0.7 | -345.6  ±2.4 | -351.7  ±0.7 | 22.0  ±1.1 | 24.6 |
| B10 | RTESEVPPRP | -48.0  ±2.7 | -54.0  ±0.6 | -6.1  ±0.0 | 92.4  ±2.2 | -37.9  ±0.2 | -580.2  ±0.9 | -582.0  ±0.2 | 24.1  ±0.9 | 26.6 |
|  |  |  |  |  |  |  |  |  |  |  |
| C1 | LASRPLPLLP | -202.8  ±5.6 | -43.2  ±0.3 | -5.9  ±0.1 | 229.2  ±5.2 | -37.1  ±0.5 | -150.5  ±1.4 | -153.7  ±0.4 | 17.6  ±3.0 | 20.1 |
| C2 | ISQRALPPLP | -189.4  ±4.7 | -49.5  ±0.3 | -6.1  ±0.0 | 226.6  ±4.2 | -45.7  ±0.6 | -215.8  ±0.4 | -216.6  ±0.6 | 28.2  ±0.3 | 30.8 |
| C3 | ITMRPLPALP | -196.6  ±8.4 | -46.9  ±0.6 | -6.1  ±0.2 | 223.4  ±7.7 | -38.4  ±0.2 | -171.5  ±2.1 | -174.0  ±1.1 | 14.7  ±1.4 | 17.3 |
| C4 | RSGRPLPPIP | -243.3  ±8.4 | -49.4  ±0.5 | -6.1  ±0.1 | 288.2  ±8.4 | -36.7  ±0.4 | -342.6  ±1.3 | -346.6  ±0.5 | 30.1  ±2.8 | 32.7 |
| C5 | KWDSLLPALP | -88.4  ±5.9 | -42.8  ±2.1 | -5.3  ±0.2 | 117.3  ±5.4 | -32.2  ±0.3 | -92.6  ±0.5 | -94.4  ±0.6 | 14.8  ±2.2 | 17.4 |
| C6 | YWDMPLPRLP | -127.3  ±7.7 | -52.8  ±1.3 | -6.0  ±0.1 | 146.2  ±5.4 | -37.2  ±0.6 | -200.4  ±0.6 | -204.7  ±1.2 | 1.6  ±0.6 | 4.2 |
| C7 | YYQRPLPPLP | -130.5  ±4.6 | -51.2  ±0.5 | -5.9  ±0.0 | 154.8  ±4.7 | -33.8  ±0.3 | -212.4  ±1.4 | -218.0  ±2.2 | 6.6  ±0.8 | 9.1 |
| C8 | YFSRALPGLP | -122.9  ±6.2 | -52.6  ±0.8 | -5.9  ±0.1 | 151.8  ±5.4 | -33.0  ±0.4 | -185.7  ±0.6 | -188.5  ±0.7 | 6.3  ±1.0 | 8.8 |
| C9 | SLWDPLPPIP | 17.4  ±6.5 | -45.2  ±0.4 | -5.4  ±0.1 | 7.5  ±6.1 | -33.7  ±0.4 | -63.4  ±1.1 | -68.1  ±0.4 | 12.6  ±1.7 | 15.2 |
| C10 | DPYDALPETP | 73.4  ±3.2 | -48.6  ±0.7 | -5.5  ±0.1 | -33.0  ±2.8 | -33.7  ±0.1 | -261.1  ±0.6 | -267.0  ±0.8 | 26.0  ±1.3 | 28.6 |
