# Supplementary material for: Computational Analysis and Prediction of the Binding Motif and Protein Interacting Partners of the Abl SH3 Domain
Source: PLoS Comput Biol. 2006 Jan 27;2(1):e1. doi: 10.1371/journal.pcbi.0020001 (PMC1356089; doi:10.1371/journal.pcbi.0020001)
Supplement: Table S2 — (56 KB DOC) [file pcbi.0020001.st002.doc]

Table S2. The energetic components and binding affinities for the 15 peptide ligands of the Abl SH3 domain using interior dielectric constant of 2 (kcal/mol).

| No. | Sequence | ΔG_exp_ | Δ*E*_ele_ | Δ*E*_vdw_ | Δ*G*_SA_ | Δ*G*_PB_ | TΔS | Glig_bound | Glig_unbound | ΔG_pred_ | ΔΔG_pred_ |
| --- | --- | --- | --- | --- | --- | --- | --- | --- | --- | --- | --- |
| A1 | APSYSPPPPP | -7.94 | -46.0  ±1.7 | -49.6  ±0.4 | -5.3  ±0.0 | 29.4  ±1.4 | -31.2  ±0.2 | 163.3  ±0.5 | 160.6  ±1.6 | -37.6  ±0.8 | 0.0 |
| A2 | APTMPPPLPP | -6.17 | -35.7  ±2.5 | -49.5  ±0.4 | -5.4  ±0.1 | 24.1  ±2.3 | -33.6  ±0.3 | 155.4  ±0.6 | 151.2  ±0.7 | -28.9  ±0.6 | 8.7 |
| A3 | PPAYPPPPVP | -7.23 | -41.6  ±1.8 | -50.8  ±0.5 | -5.4  ±0.0 | 26.3  ±1.1 | -31.8  ±0.1 | 212.7  ±0.6 | 210.4  ±0.3 | -37.4  ±0.3 | 0.2 |
| A4 | FGTYPPPLPP | -7.03 | -35.2  ±3.7 | -49.9  ±0.2 | -5.6  ±0.0 | 23.0  ±2.3 | -33.0  ±0.3 | 153.0  ±0.3 | 151.3  ±1.1 | -33.0  ±0.7 | 4.6 |
| A5 | SPSYSPPPPP | -7.83 | -50.0  ±1.8 | -48.2  ±1.0 | -5.2  ±0.0 | 30.7  ±0.6 | -31.9  ±0.1 | 149.7  ±1.0 | 149.3  ±0.5 | -40.51  ±1.0 | -2.9 |
| A6 | APTYSPPPPP | -8.72 | -51.6  ±9.5 | -50.2  ±1.6 | -5.4  ±0.1 | 31.3  ±8.2 | -31.1  ±0.4 | 145.9  ±1.5 | 142.8  ±0.5 | -41.7  ±0.5 | -4.1 |
| A7 | APTMSPPLPP | -6.37 | -40.4  ±4.2 | -50.6  ±0.8 | -5.3  ±0.0 | 27.0  ±3.2 | -33.8  ±0.3 | 137.7  ±2.2 | 137.0  ±1.6 | -34.7  ±1.0 | 2.9 |
| A8 | APTYPPPLNP | -5.48 | -48.8  ±4.0 | -51.3  ±0.5 | -5.5  ±0.0 | 30.6  ±2.7 | -31.6  ±0.4 | 128.0  ±1.2 | 122.2  ±0.1 | -37.6  ±0.2 | 0.0 |
| A9 | APTYHPPLPP | -5.30 | -37.6  ±0.7 | -51.2  ±0.5 | -5.4  ±0.0 | 25.8  ±0.6 | -30.9  ±0.1 | 166.8  ±1.9 | 161.9  ±0.7 | -32.6  ±1.2 | 5.0 |
| A10 | APTMPPPPPP | -6.95 | -40.4  ±2.5 | -48.9  ±0.6 | -5.3  ±0.0 | 26.1  ±2.3 | -33.4  ±0.4 | 155.0  ±1.2 | 154.6  ±0.1 | -34.7  ±0.4 | 2.9 |
| A11 | APTYPPPLPH | -6.23 | -41.9  ±2.1 | -53.6  ±0.7 | -5.7  ±0.0 | 28.0  ±1.7 | -32.0  ±0.2 | 164.0  ±1.9 | 154.4  ±0.7 | -31.6  ±0.8 | 6.0 |
| A12 | APTYPPPLPL | -6.17 | -43.8  ±3.3 | -51.8  ±0.4 | -5.5  ±0.1 | 28.8  ±2.8 | -31.3  ±0.4 | 141.5  ±1.3 | 136.5  ±0.2 | -36.1  ±0.4 | 1.5 |
| A13 | APTYPPPPPP | -7.36 | -46.9  ±3.6 | -50.2  ±0.5 | -5.3  ±0.1 | 29.1  ±3.3 | -31.4  ±0.2 | 157.2  ±0.7 | 156.6  ±0.9 | -41.3  ±0.8 | -3.7 |
| A14 | APTYSPPLPP | -7.23 | -45.1  ±4.8 | -51.2  ±0.3 | -5. 5  ±0.1 | 29.1  ±3.9 | -32.2  ±0.0 | 143.0  ±0.9 | 137.2  ±0.7 | -34.6  ±0.6 | 3.0 |
| A15 | ALPYPPPLPP | -5.93 | -47.6  ±0.6 | -45.7  ±0.1 | -5.1  ±0.0 | 29.8  ±0.5 | -32.9  ±0.4 | 187.7  ±0.5 | 183.8  ±1.3 | -32.8  ±0.8 | 4.8 |
