# Supplementary material for: Computational Analysis and Prediction of the Binding Motif and Protein Interacting Partners of the Abl SH3 Domain
Source: PLoS Comput Biol. 2006 Jan 27;2(1):e1. doi: 10.1371/journal.pcbi.0020001 (PMC1356089; doi:10.1371/journal.pcbi.0020001)
Supplement: Table S3 — (63 KB DOC) [file pcbi.0020001.st003.doc]

Table S3. The binding free energies for the 20 peptides mutated at position P_-5_ (kcal/mol).

| No. | Sequence | Δ*E*_ele_ | Δ*E*_vdw_ | Δ*G*_SA_ | Δ*G*_PB_ | Glig_bound | Glig_free | ΔG_pred_ | ΔΔG_pred_ |
| --- | --- | --- | --- | --- | --- | --- | --- | --- | --- |
| 1 | AASYSPPPPP | -96.4  ±5.4 | -43.8  ±0.3 | -5.0  ±0.0 | 115.1  ±5.1 | -34.2  ±0.8 | -36.9  ±0.5 | -27.4  ±1.2 | 6.4 |
| 2 | ARSYSPPPPP | -204.4  ±4.6 | -45.9  ±0.3 | -5.1  ±0.0 | 228.3  ±3.9 | -165.6  ±0.8 | -166.3  ±0.6 | -26.4  ±0.8 | 7.4 |
| 3 | ANSYSPPPPP | -95.0  ±8.1 | -43.8  ±0.4 | -4.8  ±0.0 | 110.8  ±7.9 | -82.0  ±0.3 | -83.9  ±0.8 | -30.9  ±0.7 | 2.9 |
| 4 | ADSYSPPPPP | -12.6  ±8.7 | -42.6  ±0.7 | -5.0  ±0.1 | 43.1  ±8.9 | -81.5  ±1.3 | -85.7  ±0.8 | -12.9  ±1.8 | 20.9 |
| 5 | ACSYSPPPPP | -104.9  ±5.9 | -45.1  ±1.1 | -5.3  ±0.1 | 123.2  ±5.5 | -30.0  ±0.8 | -32.6  ±1.4 | -29.4  ±0.9 | 4.4 |
| 6 | AQSYSPPPPP | -78.0  ±6.2 | -38.3  ±0.8 | -4.6  ±0.1 | 101.7  ±5.7 | -104.5  ±0.4 | -108.3  ±0.9 | -15.4  ±1.5 | 18.4 |
| 7 | AESYSPPPPP | -34.5  ±5.3 | -43.9  ±1.2 | -5.3  ±0.1 | 58.4  ±5.5 | -118.8  ±1.4 | -130.9  ±0.5 | -13.1  ±0.5 | 20.7 |
| 8 | AGSYSPPPPP | -105.4  ±3.1 | -40.3  ±1.1 | -5.2  ±0.0 | 126.0  ±2.6 | -37.7  ±1.6 | -38.0  ±0.3 | -24.5  ±0.4 | 9.3 |
| 9 | AHSYSPPPPP | -90.6  ±5.4 | -40.1  ±1.5 | -4.8  ±0.2 | 107.8  ±5.8 | -17.8  ±0.6 | -22.4  ±1.6 | -23.1  ±0.7 | 10.7 |
| 10 | AISYSPPPPP | -108.0  ±3.4 | -41.5  ±0.5 | -5.1  ±0.1 | 125.7  ±3.1 | -33.3  ±0.9 | -39.3  ±0.4 | -23.0  ±0.6 | 10.8 |
| 11 | ALSYSPPPPP | -88.7  ±14.7 | -43.2  ±0.4 | -4.9  ±0.0 | 105.6  ±14.4 | -32.5  ±1.3 | -33.3  ±1.1 | -30.3  ±1.0 | 3.5 |
| 12 | AKSYSPPPPP | -208.7  ±4.3 | -44.6  ±0.2 | -5.1  ±0.0 | 231.0  ±3.7 | -46.2  ±0.5 | -45.0  ±2.1 | -28.5  ±0.8 | 5.3 |
| 13 | AMSYSPPPPP | -97.8  ±9.4 | -44.3  ±0.3 | -5.1  ±0.0 | 116.3  ±8.6 | -41.0  ±0.9 | -41.6  ±0.4 | -30.5  ±1.0 | 3.3 |
| 14 | AFSYSPPPPP | -59.0  ±6.2 | -47.1  ±0.2 | -5.2  ±0.1 | 79.8  ±6.7 | -24.2  ±1.6 | -25.1  ±1.5 | -30.5  ±0.9 | 3.3 |
| 15 | ASSYSPPPPP | -94.6  ±6.1 | -43.4  ±0.6 | -5.1  ±0.1 | 115.9  ±5.3 | -39.1  ±1.1 | -41.3  ±0.5 | -25.0  ±0.8 | 8.8 |
| 16 | ATSYSPPPPP | -102.6  ±7.7 | -44.5  ±0.7 | -5.2  ±0.0 | 122.9  ±7.8 | -76.6  ±0.6 | -77.4  ±1.1 | -28.6  ±0.4 | 5.2 |
| 17 | AWSYSPPPPP | -83.6  ±4.5 | -44.7  ±0.6 | -5.1  ±0.0 | 109.6  ±3.5 | -12.6  ±1.4 | -16.2  ±0.5 | -20.2  ±0.3 | 13.6 |
| 18 | AYSYSPPPPP | -70.3  ±8.2 | -47.9  ±1.0 | -5.6  ±0.0 | 87.8  ±8.4 | -29.3  ±0.9 | -31.7  ±0.3 | -31.4  ±0.6 | 2.4 |
| 19 | AVSYSPPPPP | -95.2  ±4.3 | -42.7  ±0.6 | -4.8  ±0.1 | 113.1  ±4.7 | -30.7  ±0.4 | -31.4  ±0.8 | -31.8  ±0.4 | 2.0 |
| 20 | APSYSPPPPP | -92.0  ±3.4 | -49.6  ±0.4 | -5.3  ±0.0 | 112.5  ±2.9 | -17.8  ±0.6 | -18.3  ±1.5 | -33.8  ±0.7 | 0.0 |
