# Supplementary material for: Computational Analysis and Prediction of the Binding Motif and Protein Interacting Partners of the Abl SH3 Domain
Source: PLoS Comput Biol. 2006 Jan 27;2(1):e1. doi: 10.1371/journal.pcbi.0020001 (PMC1356089; doi:10.1371/journal.pcbi.0020001)
Supplement: Table S4 — (62 KB DOC) [file pcbi.0020001.st004.doc]

Table S4. The binding free energies for the 20 peptides mutated at position P_-3_ (kcal/mol)

| No. | Sequence | Δ*E*_ele_ | Δ*E*_vdw_ | Δ*G*_SA_ | Δ*G*_PB_ | Glig_bound | Glig_free | ΔG_pred_ | ΔΔG_pred_ |
| --- | --- | --- | --- | --- | --- | --- | --- | --- | --- |
| 1 | APSASPPPPP | -90.8  ±6.8 | -44.2  ±0.2 | -4.9  ±0.1 | 108.1  ±6.5 | -19.0  ±0.6 | -23.8  ±0.7 | -27.1  ±0.6 | 6.7 |
| 2 | APSRSPPPPP | -186.1  ±9.9 | -47.5  ±0.3 | -5.4  ±0.1 | 212.2  ±8.8 | -151.2  ±1.3 | -153.2  ±1.8 | -24.6  ±0.3 | 9.1 |
| 3 | APSNSPPPPP | -91.6  ±4.3 | -44.2  ±0.9 | -4.9  ±0.1 | 109.0  ±4.1 | -66.9  ±0.6 | -67.2  ±0.7 | -31.5  ±1.1 | 2.3 |
| 4 | APSDSPPPPP | -21.4  ±5.7 | -46.7  ±0.5 | -5.0  ±0.0 | 51.0  ±5.9 | -72.1  ±1.3 | -76.7  ±0.4 | -17.4  ±0.9 | 16.4 |
| 5 | APSCSPPPPP | -115.2  ±3.1 | -42.4  ±0.2 | -5.1  ±0.1 | 129.0  ±2.6 | -18.2  ±1.7 | -19.6  ±0.9 | -32.2  ±0.9 | 1.6 |
| 6 | APSQSPPPPP | -69.4  ±3.6 | -47.4  ±0.9 | -5.1  ±0.1 | 88.1  ±4.4 | -89.6  ±0.3 | -93.0  ±1.1 | -30.5  ±0.4 | 3.3 |
| 7 | APSESPPPPP | -34.9  ±10.4 | -45.1  ±1.1 | -5.0  ±0.0 | 61.1  ±9.7 | -111.3  ±0.9 | -113.2  ±1.3 | -21.8  ±0.7 | 12.0 |
| 8 | APSGSPPPPP | -99.0  ±7.9 | -43.3  ±1.3 | -4.8  ±0.1 | 115.1  ±7.6 | -25.5  ±1.1 | -27.0  ±0.2 | -30.5  ±2.1 | 3.3 |
| 9 | APSHSPPPPP | -93.3  ±5.4 | -46.2  ±1.6 | -5.1  ±0.0 | 111.5  ±5.8 | -32.3  ±0.7 | -34.9  ±0.9 | -30.5  ±0.99 | 3.3 |
| 10 | APSISPPPPP | -76.3  ±7.7 | -46.6  ±0.5 | -5.0  ±0.0 | 95.0  ±7.1 | -25.5  ±0.7 | -26.1  ±0.5 | -32.3  ±0.4 | 1.5 |
| 11 | APSLSPPPPP | -103.0  ±5.8 | -43.7  ±0.4 | -4.9  ±0.1 | 119.1  ±5.4 | -17.3  ±1.0 | -17.5  ±0.7 | -32.3  ±0.9 | 1.5 |
| 12 | APSKSPPPPP | -219.4  ±3.2 | -47.2  ±0.3 | -5.4  ±0.0 | 245.9  ±2.7 | -36.4  ±0.8 | -37.7  ±1.3 | -24.7  ±0.6 | 9.1 |
| 13 | APSMSPPPPP | -69.7  ±6.9 | -49.1  ±0.4 | -5.3  ±0.0 | 89.6  ±5.3 | -28.3  ±0.6 | -29.3  ±1.0 | -33.5  ±0.3 | 0.3 |
| 14 | APSFSPPPPP | -79.7  ±4.5 | -48.6  ±0.6 | -5.2  ±0.0 | 98.4  ±4.4 | -11.4  ±1.1 | -11.8  ±1.7 | -35.3  ±0.5 | -1.5 |
| 15 | APSPSPPPPP | -96.9  ±10.4 | -45.2  ±0.9 | -4.9  ±0.1 | 112.6  ±10.8 | 2.3  ±1.5 | 0.1  ±0.3 | -32.9  ±0.3 | 0.9 |
| 16 | APSSSPPPPP | -114.7  ±6.7 | -41.8  ±0.5 | -5.1  ±0.1 | 127.8  ±6.8 | -21.0  ±0.3 | -25.3  ±0.8 | -28.8  ±0.8 | 5.0 |
| 17 | APSTSPPPPP | -76.1  ±8.7 | -45.6  ±0.5 | -4.9  ±0.0 | 94.7  ±8.4 | -63.3  ±0.7 | -64.3  ±0.2 | -30.3  ±0.8 | 3.5 |
| 18 | APSWSPPPPP | -87.7  ±9.3 | -50.7  ±0.7 | -5.3  ±0.0 | 108.1  ±8.8 | -1.9  ±0.8 | -2.4  ±1.3 | -35.9  ±1.4 | -2.1 |
| 19 | APSVSPPPPP | -84.8  ±5.8 | -44.9  ±0.2 | -4.8  ±0.1 | 103.1  ±5.4 | -12.6  ±1.0 | -13.9  ±0.4 | -30.5  ±1.7 | 3.3 |
| 20 | APSYSPPPPP | -92.0  ±3.4 | -49.6  ±0.4 | -5.3  ±0.0 | 112.5  ±2.9 | -17.8  ±0.6 | -18.3  ±1.5 | -33.8  ±0.7 | 0.0 |
