# Supplementary material for: Computational Analysis and Prediction of the Binding Motif and Protein Interacting Partners of the Abl SH3 Domain
Source: PLoS Comput Biol. 2006 Jan 27;2(1):e1. doi: 10.1371/journal.pcbi.0020001 (PMC1356089; doi:10.1371/journal.pcbi.0020001)
Supplement: Table S5 — (61 KB DOC) [file pcbi.0020001.st005.doc]

Table S5. The binding free energies for the 20 peptides mutated at position P_0_ (kcal/mol)

| No. | Sequence | Δ*E*_ele_ | Δ*E*_vdw_ | Δ*G*_SA_ | Δ*G*_PB_ | Glig_bound | Glig_free | ΔG_pred_ | ΔΔG_pred_ |
| --- | --- | --- | --- | --- | --- | --- | --- | --- | --- |
| 1 | APSYSPAPPP | -89.5  ±7.1 | -46.2  ±0.2 | -5.2  ±0.1 | 112.5  ±7.7 | -24.9  ±0.4 | -25.8  ±0.5 | -27.5  ±1.1 | 6.3 |
| 2 | APSYSPRPPP | -142.0  ±9.5 | -47.3  ±0.5 | -5.4  ±0.1 | 175.8  ±10.1 | -150.9  ±1.1 | -153.1  ±0.5 | -16.6  ±1.3 | 17.2 |
| 3 | APSYSPNPPP | -102.8  ±4.6 | -44.7  ±1.0 | -5.3  ±0.1 | 122.2  ±4.1 | -73.4  ±0.7 | -76.2  ±0.8 | -27.6  ±0.4 | 6.2 |
| 4 | APSYSPDPPP | -50.5  ±3.6 | -48.0  ±0.8 | -5.2  ±0.0 | 81.6  ±3.3 | -82.1  ±0.3 | -81.8  ±1.4 | -22.4  ±1.3 | 11.4 |
| 5 | APSYSPCPPP | -85.2  ±6.8 | -47.0  ±0.8 | -5.3  ±0.1 | 108.8  ±5.9 | -19.7  ±0.5 | -20.8  ±1.7 | -27.5  ±0.7 | 6.3 |
| 6 | APSYSPQPPP | -95.0  ±5.5 | -48.0  ±0.3 | -5.3  ±0.1 | 122.7  ±4.7 | -95.9  ±0.6 | -99.1  ±0.9 | -22.5  ±1.0 | 11.3 |
| 7 | APSYSPEPPP | -47.5  ±4.3 | -47.3  ±0.6 | -5.2  ±0.1 | 79.5  ±3.7 | -114.3  ±1.1 | -115.3  ±0.4 | -19.6  ±0.8 | 14.2 |
| 8 | APSYSPGPPP | -79.3  ±9.2 | -46.0  ±0.4 | -5.2  ±0.0 | 102.4  ±8.8 | -32.7  ±0.8 | -33.2  ±0.7 | -27.6  ±0.5 | 6.2 |
| 9 | APSYSPHPPP | -96.9  ±5.8 | -46.2  ±0.5 | -5.2  ±0.1 | 116.4  ±6.0 | -15.4  ±0.7 | -17.5  ±1.0 | -29.8  ±0.4 | 4.0 |
| 10 | APSYSPIPPP | -95.7  ±9.9 | -47.1  ±1.2 | -5.4  ±0.1 | 121.5  ±10.2 | -24.8  ±0.8 | -24.7  ±0.3 | -26.9  ±0.9 | 6.9 |
| 11 | APSYSPLPPP | -98.4  ±7.2 | -45.2  ±0.5 | -5.1  ±0.1 | 120.7  ±7.7 | -20.4  ±0.7 | -22.1  ±0.2 | -26.3  ±1.5 | 7.5 |
| 12 | APSYSPKPPP | -133.7  ±4.7 | -48.6  ±0.5 | -5.5  ±0.1 | 168.3  ±4.5 | -37.4  ±1.1 | -44.2  ±0.5 | -12.8  ±1.4 | 21.0 |
| 13 | APSYSPMPPP | -100.3  ±7.4 | -46.3  ±0.7 | -5.2  ±0.0 | 121.5  ±6.8 | -33.3  ±1.0 | -33.6  ±0.2 | -29.8  ±0.4 | 4.0 |
| 14 | APSYSPFPPP | -97.5  ±3.3 | -47.4  ±1.1 | -5.3  ±0.1 | 120.2  ±3.1 | -16.3  ±0.5 | -20.1  ±0.7 | -26.2  ±0.8 | 7.6 |
| 15 | APSYSPSPPP | -87.4  ±3.9 | -45.7  ±0.2 | -5.2  ±0.0 | 110.9  ±4.5 | -36.0  ±0.6 | -38.9  ±0.9 | -24.4  ±0.8 | 9.4 |
| 16 | APSYSPTPPP | -92.9  ±6.2 | -48.3  ±0.6 | -5.3  ±0.1 | 117.18  ±5.9 | -61.4  ±2.1 | -61.5  ±1.5 | -29.2  ±0.9 | 4.6 |
| 17 | APSYSPWPPP | -99.2  ±4.6 | -48.7  ±0.4 | -5.3  ±0.1 | 119.11  ±4.6 | -11.6  ±1.2 | -13.2  ±0.8 | -32.4  ±1.0 | 1.4 |
| 18 | APSYSPYPPP | -113.4  ±6.4 | -43.3  ±0.9 | -5.3  ±0.1 | 131.03  ±7.2 | -24.9  ±0.6 | -28.0  ±1.3 | -28.0  ±1.3 | 5.8 |
| 19 | APSYSPVPPP | -91.4  ±10.4 | -46.9  ±1.0 | -5.3  ±0.1 | 115.31  ±11.2 | -22.1  ±1.5 | -29.1  ±0.4 | -25.3  ±1.6 | 8.5 |
| 20 | APSYSPPPPP | -92.0  ±3.7 | -49.6  ±0.4 | -5.3  ±0.0 | 112.5  ±2.9 | -17.8  ±0.6 | -18.3  ±1.5 | -33.8  ±0.7 | 0.0 |
