# Supplementary material for: Computational Analysis and Prediction of the Binding Motif and Protein Interacting Partners of the Abl SH3 Domain
Source: PLoS Comput Biol. 2006 Jan 27;2(1):e1. doi: 10.1371/journal.pcbi.0020001 (PMC1356089; doi:10.1371/journal.pcbi.0020001)
Supplement: Table S6 — (65 KB DOC) [file pcbi.0020001.st006.doc]

Table S6. The binding free energies for the 20 peptides mutated at position P_3_ (kcal/mol)

| No. | Sequence | Δ*E*_ele_ | Δ*E*_vdw_ | Δ*G*_SA_ | Δ*G*_PB_ | Glig_bound | Glig_free | TΔS | ΔG_pred_ | ΔΔG_pred_ |
| --- | --- | --- | --- | --- | --- | --- | --- | --- | --- | --- |
| 1 | APSYSPPPPA | -92.8  ±5.6 | -48.1  ±0.8 | -5.2  ±0.0 | 118.1  ±5.8 | -25.4  ±0.8 | -27.7  ±0.5 | -30.3  ±0.4 | -0.8  ±0.6 | 1.8 |
| 2 | APSYSPPPPR | -119.2  ±13.4 | -52.7  ±0.6 | -5.7  ±0.1 | 153.1  ±12.7 | -229.4  ±0.8 | -232.8  ±0.3 | -33.6  ±0.2 | 12.5  ±1.9 | 15.0 |
| 3 | APSYSPPPPN | -86.9  ±7.9 | -48.5  ±0.6 | -5.2  ±0.1 | 109.6  ±8.2 | -103.1  ±1.6 | -103.3  ±0.4 | -31.5  ±0.5 | 0.7  ±0.2 | 3.3 |
| 4 | APSYSPPPPD | -51.5  ±5.4 | -46.8  ±0.3 | -5.1  ±0.1 | 83.1  ±12.1 | -89.0  ±0.8 | -90.8  ±0.4 | -31.1  ±0.4 | 12.4  ±1.7 | 15.0 |
| 5 | APSYSPPPPC | -79.3  ±3.4 | -51.3  ±0.6 | -5.5  ±0.1 | 104.0  ±2.2 | -26.9  ±0.5 | -30.17  ±1.1 | -29.6  ±0.3 | 0.89  ±1.1 | 3.5 |
| 6 | APSYSPPPPQ | -88.6  ±7.9 | -51.3  ±0.7 | -5.5  ±0.1 | 111.4  ±9.1 | -85.1  ±0.2 | -86.6  ±1.9 | -31.0  ±0.5 | -1.2  ±0.5 | 1.3 |
| 7 | APSYSPPPPE | -71.0  ±6.4 | -50.3  ±1.1 | -5.4  ±0.0 | 100.6  ±5.7 | -82.7  ±0.7 | -84.2  ±0.6 | -32.8  ±0.5 | 8.2  ±2.2 | 10.7 |
| 8 | APSYSPPPPG | -92.4  ±9.8 | -48.0  ±1.9 | -5.2  ±0.1 | 114.4  ±8.9 | -50.8  ±0.4 | -59.0  ±0.6 | -32.1  ±0.6 | 9.1  ±1.7 | 11.6 |
| 9 | APSYSPPPPH | -98.1  ±8.1 | -52.1  ±1.2 | -5.5  ±0.0 | 121.2  ±8.4 | -29.2  ±1.1 | -31.0  ±0.5 | -31.4  ±0.2 | -1.2  ±0.6 | 1.4 |
| 10 | APSYSPPPPI | -102.4  ±6.7 | -49.8  ±0.7 | -5.4  ±0.0 | 124.8  ±5.4 | -30.9  ±1.5 | -34.5  ±0.9 | -32.2  ±0.3 | 2.9  ±1.1 | 5.5 |
| 11 | APSYSPPPPL | -104.1  ±5.5 | -48.7  ±0.3 | -5.4  ±0.0 | 124.3  ±6.6 | -48.9  ±0.5 | -50.2  ±0.4 | -32.9  ±0.5 | 0.4  ±1.4 | 3.0 |
| 12 | APSYSPPPPK | -128.8  ±8.1 | -52.4  ±0.9 | -5.7  ±0.1 | 165.6  ±7.5 | -59.1  ±0.3 | -60.6  ±0.7 | -32.4  ±0.8 | 12.7  ±1.1 | 15.2 |
| 13 | APSYSPPPPM | -94.7  ±7.2 | -51.5  ±1.1 | -5.6  ±0.1 | 117.3  ±9.1 | -32.5  ±0.7 | -34.2  ±0.6 | -30.7  ±0.6 | -2.1  ±0.7 | 0.5 |
| 14 | APSYSPPPPF | -98.1  ±6.6 | -51.6  ±1.3 | -5.5  ±0.1 | 121.1  ±7.3 | -22.6  ±0.7 | -23.8  ±0.1 | -31.1  ±0.3 | -1.9  ±0.4 | 0.7 |
| 15 | APSYSPPPPS | -100.4  ±7.6 | -47.1  ±0.1 | -5.1  ±0.2 | 121.2  ±5.8 | -50.6  ±0.2 | -53.6  ±1.3 | -32.2  ±0.2 | 3.9  ±0.9 | 6.5 |
| 16 | APSYSPPPPT | -101.9  ±3.2 | -49.1  ±0.3 | -5.3  ±0.0 | 124.6  ±1.8 | -70.9  ±0.6 | -73.5  ±0.8 | -31.7  ±0.6 | 2.7  ±0.9 | 5.2 |
| 17 | APSYSPPPPW | -97.7  ±7.9 | -52.0  ±0.4 | -5.6  ±0.1 | 121.3  ±6.5 | -21.2  ±1.3 | -21.7  ±0.5 | -32.5  ±0.5 | -0.9  ±0.5 | 1.6 |
| 18 | APSYSPPPPY | -89.7  ±3.1 | -52.9  ±0.7 | -5.6  ±0.0 | 114.0  ±4.0 | -45.4  ±0.8 | -48.0  ±1.5 | -31.6  ±0.8 | -0.1  ±0.6 | 2.5 |
| 19 | APSYSPPPPV | -88.3  ±6.8 | -49.7  ±0.2 | -5.4  ±0.1 | 110.9  ±6.2 | -60.1  ±1.5 | -62.2  ±0.8 | -31.6  ±0.4 | 1.2  ±0.6 | 3.8 |
| 20 | APSYSPPPPP | -92.0  ±3.5 | -49.6  ±0.4 | -5.3  ±0.0 | 112.5  ±2.9 | -17.8  ±0.6 | -18.3  ±1.5 | -31.2  ±0.2 | -2.6  ±0.8 | 0.0 |
