# Supplementary material for: Computational Analysis and Prediction of the Binding Motif and Protein Interacting Partners of the Abl SH3 Domain
Source: PLoS Comput Biol. 2006 Jan 27;2(1):e1. doi: 10.1371/journal.pcbi.0020001 (PMC1356089; doi:10.1371/journal.pcbi.0020001)
Supplement: Table S7 — (57 KB DOC) [file pcbi.0020001.st007.doc]

Table S7. The contribution of each residue in the template peptide APSYSPPPPP to the SH3 domain binding (kcal/mol)

| Position | SH3 | Δ*E_vdw_* | Δ*E_ele_* | Δ*E_gas_* | Δ*G_GB_* | Δ*G_SA_* | Δ*G_GB/SA_* | Δ*G_total_* |
| --- | --- | --- | --- | --- | --- | --- | --- | --- |
| P_-6_ | Total*^a^* | -1.3 | -36.6 | -38.0 | 37.2 | 34.2 | 32.7 | -5.3 |
|  | Asn15 | 0.0 | -6.2 | -6.2 | 4.9 | -0.4 | 4.6 | -1.6 |
|  | Trp47 | -0.7 | -0.6 | -1.3 | 0.1 | -0.7 | -0.7 | -2.0 |
| P_-5_ | Total*^a^* | -3.5 | -7.2 | -10.7 | 6.6 | -2.6 | 4.0 | -6.7 |
|  | Asp14 | -0.4 | -4.6 | -5.0 | 4.3 | -0.3 | 4.0 | -1.1 |
|  | Trp36 | -1.3 | -0.2 | -1.5 | 0.0 | -1.0 | -1.0 | -2.5 |
|  | Trp47 | -0.8 | -0.1 | -1.0 | 0.1 | -0.6 | -0.6 | -1.6 |
| P_-4_ | Total*^a^* | -0.1 | -1.5 | -1.6 | 1.5 | -0.1 | 1.4 | -0.2 |
| P_-3_ | Total*^a^* | -2.3 | -2.9 | -5.2 | 2.6 | -1.9 | 0.7 | -4.5 |
|  | Ser12 | -0.3 | -0.4 | -0.7 | 0.1 | -0.5 | -0.4 | -1.1 |
|  | Asp14 | -0.5 | -2.3 | -2.8 | 2.3 | -0.5 | 1.8 | -0.9 |
|  | Trp36 | -0.7 | -0.1 | -0.8 | 0.2 | -0.6 | -0.4 | -1.3 |
| P_-2_ | Total*^a^* | -0.2 | -0.9 | -1.1 | 0.9 | -0.1 | 0.7 | -0.3 |
| P_-1_ | Total*^a^* | -3.1 | -7.1 | -10.2 | 6.7 | -2.6 | 4.0 | -6.2 |
|  | Glu35 | -0.8 | -6.1 | -7.0 | 5.8 | -0.8 | 5.0 | -2.0 |
|  | Trp36 | -1.2 | -0.3 | -1.5 | 0.0 | -0.9 | -0.9 | -2.4 |
|  | Pro49 | -0.4 | 0.4 | 0.0 | -0.3 | -0.6 | -0.9 | -0.9 |
| P_0_ | Total*^a^* | -1.7 | -4.6 | -6.3 | 4.5 | -1.5 | 3.0 | -3.3 |
|  | Phe9 | -0.4 | 0.3 | -0.1 | -0.3 | -0.6 | -0.9 | -1.0 |
|  | Tyr52 | -0.5 | 0.0 | -0.5 | -0.1 | -0.5 | -0.5 | -1.0 |
| P_1_ | Total*^a^* | -0.2 | -3.5 | -3.7 | 3.5 | 0.0 | 3.5 | -0.3 |
| P_2_ | Total*^a^* | -1.7 | -3.6 | -5.4 | 3.6 | -1.5 | 2.2 | -3.3 |
|  | Tyr7 | -0.5 | -0.2 | -0.7 | 0.1 | -0.5 | -0.4 | -1.1 |
|  | Asn51 | -0.6 | -0.1 | -0.7 | 0.1 | -0.6 | -0.5 | -1.2 |
|  | Tyr52 | -0.5 | 0.2 | -0.3 | -0.2 | -0.3 | -0.6 | -0.8 |
| P_3_ | Total*^a^* | -1.5 | 16.9 | 15.3 | -16.9 | -1.4 | -18.2 | -2.9 |
|  | Tyr7 | -1.0 | -1.1 | -2.1 | 0.9 | -1.1 | -0.2 | -2.3 |

*^a^*Total represents the interactions of residue at this position with the all residues of SH3
