# Supplementary material for: Computational Analysis and Prediction of the Binding Motif and Protein Interacting Partners of the Abl SH3 Domain
Source: PLoS Comput Biol. 2006 Jan 27;2(1):e1. doi: 10.1371/journal.pcbi.0020001 (PMC1356089; doi:10.1371/journal.pcbi.0020001)
Supplement: Table S8 — (54 KB DOC) [file pcbi.0020001.st008.doc]

Table S8. The binding peptides of the Abl SH3 domain identified by SPMFEP, Scansite and iSPOT in the 13 Abl SH3-binding proteins

| Protein | SPMFEP*^a^* | | | Scansite*^b^* | | | iSPOT*^c^* | | |
| --- | --- | --- | --- | --- | --- | --- | --- | --- | --- |
|  | Peptide | Position | rank | Peptide | Position | rank | Peptide | Position | rank |
| 3BP1_mouse (7) | APTMPPPLPP  PPLPPVPPQP | 528-537  533-542 | 1393  1395 |  |  |  | PPPALPPQPP*^d^* | 583-592 | 347 |
| 3BP2_mouse (8) | TYPPAYPPPP  PPAYPPPPVP | 199-208  201-210 | 1864  2034 | PPAYPPPPVP  PPTSEPPPVP | 201-210  343-352 | 7  1053 | PPAYPPPPVP | 201-210 | 21 |
| EVL_human (9) | PPPPPVPPPP  PPPPPPPVPP  PPPPPPPPVP | 185-194  183-192  182-191 | 8  119  563 | PTGATPPPPP  PPPPPPPPVP  PPPPPPPVPP | 194-203  182-191  183-192 | 83  233  372 | PPPPPPPPVP | 182-191 | 225 |
| NMDE4_human(10) | FPGFPSPPAP | 1024-1033 | 19 | GGWWAPPPPP | 1204-1213 | 429 |  |  |  |
| P73_human (11) |  |  |  | AFKQSPPAVP | 329-338 | 321 | PPSYGPVLSP*^e^* |  | 1634 |
| PLS1_human (12) | GPQVSYPPPP | 41-50 | 533 |  |  |  |  |  |  |
| WASF1_human (13) | TPPPPVPPPP  TPVFVSPTPP  SPTPPPPPPP  APGPHVPLMP  PPPPPPPLPP  PPVPPPPPPP  TPPPPPPPPP  PPPPPPPPLP | 347-356  316-325  321-330  467-471  425-434  350-359  323-332  424-433 | 9  63  289  340  407  425  493  494 | PPPVPPPPPP  MTSTPPPPVP | 349-358  364-353 | 132  449 | PPPVPPPPPP | 349-358 | 39 |
| PDE4D_human (14) | PPPPPSPQPQ  LPPPPPPSPQ  PPPLPPPPPP  QPPPPPPPPP | 59-68  57-66  78-87  74-83 | 71  122  306  398 | PPPLPPPPPP  CPLQPPPPPP  PPPPPLPPPP | 78-87  71-80  86-85 | 127  440  508 | PPPLPPPPPP | 78-87 | 43 |
| Abi1_human (15) | SPTPPPPPPP | 392-401 | 288 | PPSGAPPAPP | 280-289 | 27 |  |  |  |
| CABL2_mouse (16) |  |  |  |  |  |  |  |  |  |
| ENAH_mouse (17) | PPPPPPPAPP  SPLPPSPPIM  APPPPPPPPP  PPPPPPPPPP  PPPPPPPPPP  PPPPPPPPPP  PPPPPPPPPP  PPPPPPPPPP  PPPPPPPPPP  GPPPPPPPPP  PPPPPPPPP  PPPPGPPPPP  PPPPPPPLPP  PPPPPPPPLP  PPPPPPPPLP | 594-603  378-387  441-450  442-451  443-452  444-453  445-454  446-455  447-456  577-586  578-587  562-571  450-459  449-458  580-589 | 398  430  651  652  653  654  655  656  657  658  659  1434  1627  1968  1969 | PPPGPPPPPP  GPAAPPPPPP  PPPLPPPPLP  LPNQAPPPPP  PPPPPPPPPP  PPPPPPPPPP  GPPAPPPPPP  LPSTGPPPPP  PPPPPPPPPP  PPPPPPPPPP  PPPPPPPPPP  PPPPPPPPPPP  PSTGPPPPPP  GPPPPPPPPP  PPPPPPPAPP | 563-572  438-447  454-463  588-597  446-455  443-452  541-550  573-582  447-456  442-451  444-453  445-454  574-583  577-586  594-603 | 137  257  318  321  358  619  829  834  880  1257  1258  1259  1260  1474  1774 |  |  | √*^f^* |
| SEM6D_mouse (18) |  |  |  |  |  |  |  |  |  |
| CRK_human (19) | PPVPPSPAQP | 69-78 | 50 |  |  |  |  |  |  |

*^a^*For the five mouse proteins, the potential binding peptides should be in the top 2000 peptides found in all proteins. For the eight human proteins, the potential binding peptides should be in the top 600 peptides found in human proteins. *^b^*For the five mouse proteins, the potential binding peptides should be in the top 2000 peptides found in all proteins. For the eight human proteins, the potential binding peptides should be in the top 600 peptides found in human proteins. *^c^*The potential binding peptides should be in the top 2000 peptides found in all proteins because iSPOT cannot give separate searching based on selected protein of specific species. *^d^*PPPALPPQPP is not the correct binding peptide segment according to reference 7 and the actual binding segment is APTMPPPLPP. *^e^*PPSYGPVLSP is not the correct binding peptide segment according to reference 11 and the actual binding segment is AFKQSPPAVP. *^f^*This protein can not be found in iSPOT high-rank list, but we think that this protein can be identified by iSPOT because several peptide segments in this protein have high iSPOT scores.
