# Supplementary material for: Computational Analysis and Prediction of the Binding Motif and Protein Interacting Partners of the Abl SH3 Domain
Source: PLoS Comput Biol. 2006 Jan 27;2(1):e1. doi: 10.1371/journal.pcbi.0020001 (PMC1356089; doi:10.1371/journal.pcbi.0020001)
Supplement: Table S9 — (643 KB DOC) [file pcbi.0020001.st009.doc]

Table S9. The top 600 ten-residue-long peptide sequences found in the human proteome in SWISS-PROT using SPMFEP.

| Rank | Protein | First | Last | Peptide | Score |
| --- | --- | --- | --- | --- | --- |
| 1 | RW1_HUMAN | 1521 | 1530 | SPTPASPSPP | -4.1 |
| 2 | WASF4_HUMAN | 475 | 484 | PPPPSSPSFP | -3.6 |
| 3 | TREX1_HUMAN | 107 | 116 | GPPPTVPPPP | -3.4 |
| 4 | ACRO_HUMAN | 344 | 353 | PPPPPSPPPP | -3.2 |
| 5 | LRRN5_HUMAN | 22 | 31 | VVPWHVPCPP | -2.9 |
| 6 | SEM6A_HUMAN | 791 | 800 | MPPMGSPVIP | -2.9 |
| 7 | HDAC4_HUMAN | 343 | 352 | LPLYTSPSLP | -2.8 |
| 8 | EVL_HUMAN | 185 | 194 | PPPPPVPPPP | -2.6 |
| 9 | WASF1_HUMAN | 347 | 356 | TPPPPVPPPP | -2.6 |
| 10 | YLPM1_HUMAN | 14 | 23 | YPPPPVPPPP | -2.6 |
| 11 | MLL4_HUMAN | 438 | 447 | PPPLPSPPPP | -2.6 |
| 12 | MRTFA_HUMAN | 802 | 811 | PPPPGSPSLP | -2.5 |
| 13 | FBSH_HUMAN | 126 | 135 | PPAFASPPDP | -2.5 |
| 14 | STF1_HUMAN | 123 | 132 | GPPMGVPPPP | -2.5 |
| 15 | PEPP2_HUMAN | 246 | 255 | MPPFPPPSLP | -2.5 |
| 16 | AF4_HUMAN | 937 | 946 | ANPFPVPSLP | -2.4 |
| 17 | WBP2_HUMAN | 167 | 176 | PPGYPYPPPP | -2.4 |
| 18 | LAP4_HUMAN | 1432 | 1441 | QPPWASPSPT | -2.3 |
| 19 | NMDE4_HUMAN | 1024 | 1033 | FPGFPSPPAP | -2.3 |
| 20 | PDCD7_HUMAN | 29 | 38 | PPPLPSPAFP | -2.2 |
| 21 | MKRN2_HUMAN | 75 | 84 | SPAFHSPHPP | -2.2 |
| 22 | DYR1B_HUMAN | 552 | 561 | PPSPTSPPPP | -2.2 |
| 23 | ACROL_HUMAN | 165 | 174 | PPPPPSPLPP | -2.2 |
| 24 | JPH2_HUMAN | 187 | 196 | GPALPSPAIP | -2.1 |
| 25 | PPAL_HUMAN | 220 | 229 | LPPWASPQTM | -2.1 |
| 26 | ACRO_HUMAN | 354 | 363 | PPPPASPLPP | -2 |
| 27 | ZN335_HUMAN | 741 | 750 | GPPPSSPGPP | -2 |
| 28 | CD2L7_HUMAN | 529 | 538 | LPTIASPPPP | -2 |
| 29 | ATF5_HUMAN | 185 | 194 | QPPPPSPPQP | -1.9 |
| 30 | CADH7_HUMAN | 374 | 383 | PPVFSSPLYP | -1.8 |
| 31 | SOX3_HUMAN | 284 | 293 | PPSMSSPPPP | -1.8 |
| 32 | E41LB_HUMAN | 826 | 835 | CPGPTSPLIP | -1.8 |
| 33 | DAG1_HUMAN | 339 | 348 | VPTPTSPAIA | -1.8 |
| 34 | K1H6_HUMAN | 423 | 432 | PPVPCVPSVP | -1.6 |
| 35 | KCC2B_HUMAN | 430 | 439 | APACPSPAPF | -1.6 |
| 36 | KPCD2_HUMAN | 14 | 23 | SPGPGSPPPP | -1.6 |
| 37 | NCOR2_HUMAN | 809 | 818 | PPAPPSPSAP | -1.5 |
| 38 | NFIB_HUMAN | 359 | 368 | TPPPPSPLPF | -1.5 |
| 39 | B3GT4_HUMAN | 41 | 50 | LPAPASPGPP | -1.5 |
| 40 | RIN1_HUMAN | 262 | 271 | VPPPPVPVLP | -1.5 |
| 41 | PTN5_HUMAN | 32 | 41 | MPPPPPPSPP | -1.4 |
| 42 | WNK4_HUMAN | 834 | 843 | PPCHPSPSPF | -1.4 |
| 43 | DRD4_HUMAN | 240 | 249 | GPGPPSPTPP | -1.4 |
| 44 | SRBP1_HUMAN | 160 | 169 | TPVLGYPSPP | -1.3 |
| 45 | GNDS_HUMAN | 362 | 371 | QPSWPSPVVA | -1.3 |
| 46 | RBM12_HUMAN | 218 | 227 | PPVPPVPPIP | -1.2 |
| 47 | KCC2B_HUMAN | 515 | 524 | PPPCPSPTIP | -1.2 |
| 48 | DOCK6_HUMAN | 328 | 337 | SVTYPSPDIF | -1.1 |
| 49 | RTN1_HUMAN | 441 | 450 | PPSPASPSIQ | -1.1 |
| 50 | CRK_HUMAN | 69 | 78 | PPVPPSPAQP | -1.1 |
| 51 | GRIK5_HUMAN | 10 | 19 | IVAFASPSCQ | -1.1 |
| 52 | ODO2_HUMAN | 184 | 193 | MPPVPSPSQP | -1 |
| 53 | ATBF1_HUMAN | 3126 | 3135 | LPGLNSPSLP | -1 |
| 54 | NFASC_HUMAN | 165 | 174 | PPGLPSPVIF | -1 |
| 55 | CT065_HUMAN | 95 | 104 | SPCLPSPDIW | -1 |
| 56 | FMOD_HUMAN | 62 | 71 | AYTYGSPSPP | -1 |
| 57 | ROBO1_HUMAN | 1480 | 1489 | LPPPPVPPPA | -0.9 |
| 58 | UBQL3_HUMAN | 474 | 483 | PPWLPSPAYP | -0.9 |
| 59 | CORO7_HUMAN | 770 | 779 | CNSFTSPDPH | -0.9 |
| 60 | WBP2_HUMAN | 195 | 204 | PPPPPYPGPM | -0.9 |
| 61 | ATF5_HUMAN | 119 | 128 | APPLPPPSPP | -0.8 |
| 62 | GCP60_HUMAN | 36 | 45 | PPPLPPPSPP | -0.8 |
| 63 | WASF1_HUMAN | 316 | 325 | TPVFVSPTPP | -0.8 |
| 64 | CR1_HUMAN | 1401 | 1410 | QFPFASPTIP | -0.8 |
| 65 | BAT2_HUMAN | 663 | 672 | TPVPPSPPQP | -0.7 |
| 66 | PCD15_HUMAN | 1778 | 1787 | CPPPSPPSIP | -0.7 |
| 67 | RBM12_HUMAN | 224 | 233 | PPIPPVPSVP | -0.7 |
| 68 | NU214_HUMAN | 1973 | 1982 | APVFGSPPTF | -0.7 |
| 69 | MCRS1_HUMAN | 103 | 112 | TPVPPSPAPA | -0.7 |
| 70 | SYTL1_HUMAN | 154 | 163 | GPDFPSPSVP | -0.7 |
| 71 | PDE4D_HUMAN | 59 | 68 | PPPPPSPQPQ | -0.7 |
| 72 | PAXI_HUMAN | 44 | 53 | AVPPPVPPPP | -0.7 |
| 73 | SCTR_HUMAN | 260 | 269 | AFGWGSPAIF | -0.6 |
| 74 | ZIC5_HUMAN | 133 | 142 | LPPTPSPPPP | -0.6 |
| 75 | NFC1_HUMAN | 841 | 850 | SLCPSSPSPP | -0.6 |
| 76 | SYT5_HUMAN | 7 | 16 | TPGPPSPDTP | -0.6 |
| 77 | BRD4_HUMAN | 777 | 786 | PPPPPPPSMP | -0.6 |
| 78 | WASP_HUMAN | 389 | 398 | GPPMPPPPPP | -0.5 |
| 79 | GPAA1_HUMAN | 408 | 417 | APGPSVPLPP | -0.5 |
| 80 | MBD6_HUMAN | 835 | 844 | APPHGSPDPP | -0.5 |
| 81 | RRB_HUMAN | 72 | 81 | VPSPPSPLPP | -0.5 |
| 82 | SC24C_HUMAN | 267 | 276 | LPPMHSPQQP | -0.5 |
| 83 | PCD15_HUMAN | 1750 | 1759 | PPPISPPSPP | -0.5 |
| 84 | EP400_HUMAN | 2245 | 2254 | MPLWTPPTPP | -0.5 |
| 85 | ULK1_HUMAN | 287 | 296 | SPPVPVPSYP | -0.5 |
| 86 | EP400_HUMAN | 310 | 319 | GFGMTSPPPP | -0.4 |
| 87 | FXL19_HUMAN | 274 | 283 | GPAVPSPSPQ | -0.4 |
| 88 | Y0355_HUMAN | 691 | 700 | QPSLPVPPPP | -0.4 |
| 89 | RPOM_HUMAN | 726 | 735 | CPQLGVPAPP | -0.4 |
| 90 | JUND_HUMAN | 219 | 228 | PVPFPPPPPP | -0.3 |
| 91 | BPA1_HUMAN | 1137 | 1146 | SVCFTVPPPN | -0.3 |
| 92 | BPAEA_HUMAN | 610 | 619 | SVCFTVPPPN | -0.3 |
| 93 | BPAEB_HUMAN | 610 | 619 | SVCFTVPPPN | -0.3 |
| 94 | SYNJ1_HUMAN | 1540 | 1549 | PPPPPVPLLP | -0.3 |
| 95 | CXX1_HUMAN | 120 | 129 | LPPLPSPLLP | -0.3 |
| 96 | E2F4_HUMAN | 202 | 211 | SPPVAVPVPP | -0.3 |
| 97 | SMF_HUMAN | 163 | 172 | AYCYTSPGPP | -0.3 |
| 98 | WASF2_HUMAN | 347 | 356 | PPPPSPPSFP | -0.3 |
| 99 | TTY7_HUMAN | 111 | 120 | APLWRSPSWP | -0.3 |
| 100 | MGR1_HUMAN | 1142 | 1151 | SPALTPPSPF | -0.3 |
| 101 | Y0310_HUMAN | 1089 | 1098 | VPMFPVPLPP | -0.3 |
| 102 | DIAP2_HUMAN | 561 | 570 | GPPPPPPAPP | -0.3 |
| 103 | ENAH_HUMAN | 362 | 371 | PPPPPPPAPP | -0.3 |
| 104 | HCN2_HUMAN | 22 | 31 | PPPPPPPAPP | -0.3 |
| 105 | HCN2_HUMAN | 35 | 44 | PPPPPPPAPP | -0.3 |
| 106 | PROM1_HUMAN | 856 | 865 | NPVMTSPSQH | -0.3 |
| 107 | K1093_HUMAN | 54 | 63 | SPIGSSPSPP | -0.2 |
| 108 | CEBPA_HUMAN | 227 | 236 | PPPTPVPSPH | -0.2 |
| 109 | ATBF1_HUMAN | 2068 | 2077 | APPITSPTIA | -0.2 |
| 110 | OR4FE_HUMAN | 257 | 266 | VYTWPSPSTH | -0.2 |
| 111 | SC24A_HUMAN | 272 | 281 | SVGYSYPSLP | -0.2 |
| 112 | BTBD2_HUMAN | 54 | 63 | APGPTPPAPP | -0.2 |
| 113 | ESX1L_HUMAN | 244 | 253 | PPVLPVPPMP | -0.2 |
| 114 | GPR57_HUMAN | 153 | 162 | AFCWSVPALF | -0.1 |
| 115 | PLS3_HUMAN | 13 | 22 | SPPPPYPVTP | -0.1 |
| 116 | OR4C3_HUMAN | 50 | 59 | SPTLASPVYF | -0.1 |
| 117 | IL21R_HUMAN | 527 | 536 | PPPLSSPGPQ | -0.1 |
| 118 | DVL2_HUMAN | 685 | 694 | MPPPPPPVPP | -0.1 |
| 119 | EVL_HUMAN | 183 | 192 | PPPPPPPVPP | -0.1 |
| 120 | RNF38_HUMAN | 238 | 247 | LPVCSVPPPM | -0.1 |
| 121 | GTR6_HUMAN | 55 | 64 | ALVYTSPVIP | -0.1 |
| 122 | PDE4D_HUMAN | 57 | 66 | LPPPPPPSPQ | -0.1 |
| 123 | PO6F1_HUMAN | 88 | 97 | QPTPTVPQPA | 0 |
| 124 | MUC5B_HUMAN | 5678 | 5687 | CTPFCVPAPM | 0 |
| 125 | RIN3_HUMAN | 309 | 318 | CPLPTSPPVP | 0 |
| 126 | FOSL1_HUMAN | 232 | 241 | SLVFTYPSTP | 0 |
| 127 | IGHA2_HUMAN | 98 | 107 | TVPCPVPPPP | 0 |
| 128 | LAF4_HUMAN | 562 | 571 | APPPAVPCAP | 0 |
| 129 | SEM5A_HUMAN | 387 | 396 | QPVTTVPSFM | 0 |
| 130 | HES2_HUMAN | 155 | 164 | APVPSPPSPP | 0.1 |
| 131 | K1002_HUMAN | 668 | 677 | SPSPCSPPQM | 0.1 |
| 132 | FANCC_HUMAN | 340 | 349 | YFPYTSPSLA | 0.1 |
| 133 | HME1_HUMAN | 51 | 60 | SPQPAPPSPP | 0.1 |
| 134 | ACRO_HUMAN | 361 | 370 | LPPPPPPPPP | 0.1 |
| 135 | ACROL_HUMAN | 160 | 169 | SPPPPPPPPP | 0.1 |
| 136 | ACROL_HUMAN | 172 | 181 | LPPPPPPPPP | 0.1 |
| 137 | ATBF1_HUMAN | 2039 | 2048 | PPPPPPPPPP | 0.1 |
| 138 | ATBF1_HUMAN | 2040 | 2049 | PPPPPPPPPP | 0.1 |
| 139 | ATBF1_HUMAN | 2041 | 2050 | PPPPPPPPPP | 0.1 |
| 140 | BAI1_HUMAN | 1410 | 1419 | QPPPPPPPPP | 0.1 |
| 141 | BAI1_HUMAN | 1411 | 1420 | PPPPPPPPPP | 0.1 |
| 142 | BAI1_HUMAN | 1412 | 1421 | PPPPPPPPPP | 0.1 |
| 143 | BAI1_HUMAN | 1413 | 1422 | PPPPPPPPPP | 0.1 |
| 144 | BAT3_HUMAN | 656 | 665 | APPPPPPPPP | 0.1 |
| 145 | BAT3_HUMAN | 657 | 666 | PPPPPPPPPP | 0.1 |
| 146 | BAT3_HUMAN | 658 | 667 | PPPPPPPPPP | 0.1 |
| 147 | BAT3_HUMAN | 659 | 668 | PPPPPPPPPP | 0.1 |
| 148 | CCDC6_HUMAN | 439 | 448 | QPPPPPPPPP | 0.1 |
| 149 | CD2L7_HUMAN | 1271 | 1280 | GPPPPPPPPP | 0.1 |
| 150 | CEBPB_HUMAN | 161 | 170 | HPPPPPPPPP | 0.1 |
| 151 | DDX17_HUMAN | 638 | 647 | YPPPPPPPPP | 0.1 |
| 152 | DIAP1_HUMAN | 598 | 607 | TPPPPPPPPP | 0.1 |
| 153 | DIAP1_HUMAN | 599 | 608 | PPPPPPPPPP | 0.1 |
| 154 | DIAP1_HUMAN | 600 | 609 | PPPPPPPPPP | 0.1 |
| 155 | DIAP1_HUMAN | 601 | 610 | PPPPPPPPPP | 0.1 |
| 156 | DIAP1_HUMAN | 602 | 611 | PPPPPPPPPP | 0.1 |
| 157 | DIAP3_HUMAN | 330 | 339 | VPPPPPPPPP | 0.1 |
| 158 | DIAP3_HUMAN | 331 | 340 | PPPPPPPPPP | 0.1 |
| 159 | DIAP3_HUMAN | 332 | 341 | PPPPPPPPPP | 0.1 |
| 160 | DOT1L_HUMAN | 1592 | 1601 | PPPPPPPPPP | 0.1 |
| 161 | DOT1L_HUMAN | 1593 | 1602 | PPPPPPPPPP | 0.1 |
| 162 | ENAH_HUMAN | 346 | 355 | GPPPPPPPPP | 0.1 |
| 163 | ENL_HUMAN | 271 | 280 | GPPPPPPPPP | 0.1 |
| 164 | GABR2_HUMAN | 11 | 20 | GPPPPPPPPP | 0.1 |
| 165 | HD_HUMAN | 40 | 49 | QPPPPPPPPP | 0.1 |
| 166 | HD_HUMAN | 41 | 50 | PPPPPPPPPP | 0.1 |
| 167 | HD_HUMAN | 42 | 51 | PPPPPPPPPP | 0.1 |
| 168 | HD_HUMAN | 68 | 77 | QPPPPPPPPP | 0.1 |
| 169 | HD_HUMAN | 69 | 78 | PPPPPPPPPP | 0.1 |
| 170 | HXB4_HUMAN | 72 | 81 | GPPPPPPPPP | 0.1 |
| 171 | HXB4_HUMAN | 73 | 82 | PPPPPPPPPP | 0.1 |
| 172 | HXB4_HUMAN | 74 | 83 | PPPPPPPPPP | 0.1 |
| 173 | HXB4_HUMAN | 75 | 84 | PPPPPPPPPP | 0.1 |
| 174 | HXB4_HUMAN | 76 | 85 | PPPPPPPPPP | 0.1 |
| 175 | HXB4_HUMAN | 77 | 86 | PPPPPPPPPP | 0.1 |
| 176 | HXB4_HUMAN | 78 | 87 | PPPPPPPPPP | 0.1 |
| 177 | HXD8_HUMAN | 114 | 123 | HPPPPPPPPP | 0.1 |
| 178 | M3K4_HUMAN | 25 | 34 | PPPPPPPPPP | 0.1 |
| 179 | M3K4_HUMAN | 26 | 35 | PPPPPPPPPP | 0.1 |
| 180 | M3K4_HUMAN | 27 | 36 | PPPPPPPPPP | 0.1 |
| 181 | NRX2B_HUMAN | 22 | 31 | LPPPPPPPPP | 0.1 |
| 182 | NRX2B_HUMAN | 23 | 32 | PPPPPPPPPP | 0.1 |
| 183 | OST4_HUMAN | 6 | 15 | APPPPPPPPP | 0.1 |
| 184 | OST4_HUMAN | 7 | 16 | PPPPPPPPPP | 0.1 |
| 185 | P2BB_HUMAN | 10 | 19 | APPPPPPPPP | 0.1 |
| 186 | P2BB_HUMAN | 11 | 20 | PPPPPPPPPP | 0.1 |
| 187 | P2BB_HUMAN | 12 | 21 | PPPPPPPPPP | 0.1 |
| 188 | PCD15_HUMAN | 1434 | 1443 | APPPPPPPPP | 0.1 |
| 189 | PCLO_HUMAN | 2335 | 2344 | QPPPPPPPPP | 0.1 |
| 190 | PCLO_HUMAN | 2336 | 2345 | PPPPPPPPPP | 0.1 |
| 191 | PCLO_HUMAN | 2337 | 2346 | PPPPPPPPPP | 0.1 |
| 192 | PCLO_HUMAN | 2338 | 2347 | PPPPPPPPPP | 0.1 |
| 193 | PCLO_HUMAN | 2339 | 2348 | PPPPPPPPPP | 0.1 |
| 194 | PCLO_HUMAN | 2340 | 2349 | PPPPPPPPPP | 0.1 |
| 195 | PCLO_HUMAN | 2341 | 2350 | PPPPPPPPPP | 0.1 |
| 196 | PCLO_HUMAN | 2342 | 2351 | PPPPPPPPPP | 0.1 |
| 197 | PCLO_HUMAN | 2343 | 2352 | PPPPPPPPPP | 0.1 |
| 198 | PCLO_HUMAN | 2344 | 2353 | PPPPPPPPPP | 0.1 |
| 199 | PCLO_HUMAN | 2345 | 2354 | PPPPPPPPPP | 0.1 |
| 200 | PCLO_HUMAN | 2346 | 2355 | PPPPPPPPPP | 0.1 |
| 201 | PCLO_HUMAN | 2347 | 2356 | PPPPPPPPPP | 0.1 |
| 202 | PCLO_HUMAN | 2348 | 2357 | PPPPPPPPPP | 0.1 |
| 203 | PRIMA_HUMAN | 60 | 69 | LPPPPPPPPP | 0.1 |
| 204 | PRIMA_HUMAN | 61 | 70 | PPPPPPPPPP | 0.1 |
| 205 | R3HDM_HUMAN | 464 | 473 | HPPPPPPPPP | 0.1 |
| 206 | R3HDM_HUMAN | 465 | 474 | PPPPPPPPPP | 0.1 |
| 207 | R3HDM_HUMAN | 466 | 475 | PPPPPPPPPP | 0.1 |
| 208 | R3HDM_HUMAN | 467 | 476 | PPPPPPPPPP | 0.1 |
| 209 | R3HDM_HUMAN | 468 | 477 | PPPPPPPPPP | 0.1 |
| 210 | RANB9_HUMAN | 81 | 90 | APPPPPPPPP | 0.1 |
| 211 | RANB9_HUMAN | 82 | 91 | PPPPPPPPPP | 0.1 |
| 212 | RANB9_HUMAN | 83 | 92 | PPPPPPPPPP | 0.1 |
| 213 | SALL2_HUMAN | 159 | 168 | APPPPPPPPP | 0.1 |
| 214 | SALL2_HUMAN | 160 | 169 | PPPPPPPPPP | 0.1 |
| 215 | SALL2_HUMAN | 161 | 170 | PPPPPPPPPP | 0.1 |
| 216 | SEM4G_HUMAN | 764 | 773 | APPPPPPPPP | 0.1 |
| 217 | SEM4G_HUMAN | 765 | 774 | PPPPPPPPPP | 0.1 |
| 218 | SF01_HUMAN | 581 | 590 | APPPPPPPPP | 0.1 |
| 219 | SF3B2_HUMAN | 80 | 89 | QPPPPPPPPP | 0.1 |
| 220 | SF3B2_HUMAN | 81 | 90 | PPPPPPPPPP | 0.1 |
| 221 | SMN_HUMAN | 216 | 225 | GPPPPPPPPP | 0.1 |
| 222 | SMN_HUMAN | 217 | 226 | PPPPPPPPPP | 0.1 |
| 223 | SN1L2_HUMAN | 822 | 831 | QPPPPPPPPP | 0.1 |
| 224 | SN1L2_HUMAN | 823 | 832 | PPPPPPPPPP | 0.1 |
| 225 | TRRAP_HUMAN | 505 | 514 | APPPPPPPPP | 0.1 |
| 226 | TRRAP_HUMAN | 506 | 515 | PPPPPPPPPP | 0.1 |
| 227 | UBP51_HUMAN | 130 | 139 | PPPPPPPPPP | 0.1 |
| 228 | UBP51_HUMAN | 131 | 140 | PPPPPPPPPP | 0.1 |
| 229 | WASF2_HUMAN | 390 | 399 | APPPPPPPPP | 0.1 |
| 230 | WASF2_HUMAN | 391 | 400 | PPPPPPPPPP | 0.1 |
| 231 | WASF4_HUMAN | 518 | 527 | APPPPPPPPP | 0.1 |
| 232 | WASL_HUMAN | 372 | 381 | APPPPPPPPP | 0.1 |
| 233 | WASL_HUMAN | 373 | 382 | PPPPPPPPPP | 0.1 |
| 234 | WASL_HUMAN | 374 | 383 | PPPPPPPPPP | 0.1 |
| 235 | WASP_HUMAN | 392 | 401 | MPPPPPPPPP | 0.1 |
| 236 | WASP_HUMAN | 393 | 402 | PPPPPPPPPP | 0.1 |
| 237 | WASP_HUMAN | 394 | 403 | PPPPPPPPPP | 0.1 |
| 238 | WT1_HUMAN | 59 | 68 | APPPPPPPPP | 0.1 |
| 239 | ZIC5_HUMAN | 138 | 147 | SPPPPPPPPP | 0.1 |
| 240 | ZIC5_HUMAN | 139 | 148 | PPPPPPPPPP | 0.1 |
| 241 | ZIC5_HUMAN | 140 | 149 | PPPPPPPPPP | 0.1 |
| 242 | ZIC5_HUMAN | 385 | 394 | LPPPPPPPPP | 0.1 |
| 243 | ZIC5_HUMAN | 386 | 395 | PPPPPPPPPP | 0.1 |
| 244 | ZIC5_HUMAN | 387 | 396 | PPPPPPPPPP | 0.1 |
| 245 | ZIC5_HUMAN | 388 | 397 | PPPPPPPPPP | 0.1 |
| 246 | ZIC5_HUMAN | 389 | 398 | PPPPPPPPPP | 0.1 |
| 247 | ZIC5_HUMAN | 390 | 399 | PPPPPPPPPP | 0.1 |
| 248 | ZIC5_HUMAN | 391 | 400 | PPPPPPPPPP | 0.1 |
| 249 | 3BP1_HUMAN | 646 | 655 | SPGPASPSPV | 0.1 |
| 250 | MY18B_HUMAN | 20 | 29 | SPPPSSPPPL | 0.1 |
| 251 | IGHA1_HUMAN | 106 | 115 | TPPTPSPSTP | 0.2 |
| 252 | MLL4_HUMAN | 418 | 427 | PPPSTSPPPP | 0.2 |
| 253 | CXX1_HUMAN | 28 | 37 | WPPPGSPPAP | 0.2 |
| 254 | NOCT_HUMAN | 405 | 414 | LPSFNYPSDH | 0.2 |
| 255 | SHAN1_HUMAN | 1205 | 1214 | SPVPPSPSPV | 0.2 |
| 256 | FOXD1_HUMAN | 81 | 90 | PPAGGSPAPP | 0.2 |
| 257 | ZP1_HUMAN | 390 | 399 | IFPPPSPAPM | 0.3 |
| 258 | EPAS1_HUMAN | 454 | 463 | LPAFTVPQAA | 0.3 |
| 259 | RADI_HUMAN | 471 | 480 | PPPPPPPVIP | 0.3 |
| 260 | WASF3_HUMAN | 344 | 353 | PPPPPPPVIP | 0.3 |
| 261 | ETV7_HUMAN | 134 | 143 | TPTQHSPVPP | 0.3 |
| 262 | SC24B_HUMAN | 259 | 268 | TLTWSSPGLP | 0.3 |
| 263 | DYN3_HUMAN | 786 | 795 | APAIPSPGPH | 0.3 |
| 264 | KR124_HUMAN | 57 | 66 | QPACCVPSPC | 0.3 |
| 265 | ZFHX2_HUMAN | 192 | 201 | APLFTPPVLP | 0.3 |
| 266 | CTDSL_HUMAN | 57 | 66 | APPPSSPSVL | 0.3 |
| 267 | EGFL4_HUMAN | 2068 | 2077 | QPPPAPPPPP | 0.3 |
| 268 | MLL4_HUMAN | 620 | 629 | PPPPAPPPPP | 0.3 |
| 269 | NSD1_HUMAN | 2221 | 2230 | YVPPPVPLPP | 0.3 |
| 270 | SHAN2_HUMAN | 981 | 990 | IPPPAPPPPP | 0.3 |
| 271 | WASF2_HUMAN | 317 | 326 | APPPAPPPPP | 0.3 |
| 272 | WASF4_HUMAN | 445 | 454 | APPPAPPPPP | 0.3 |
| 273 | WASIP_HUMAN | 4 | 13 | PPPPAPPPPP | 0.3 |
| 274 | WBS14_HUMAN | 386 | 395 | LPPPPVPPPL | 0.3 |
| 275 | WT1_HUMAN | 55 | 64 | APPPAPPPPP | 0.3 |
| 276 | ZIC5_HUMAN | 331 | 340 | APPPAPPPPP | 0.3 |
| 277 | GPNMB_HUMAN | 321 | 330 | GPCPPPPPPP | 0.3 |
| 278 | IGHD_HUMAN | 342 | 351 | VPAPPSPQPA | 0.3 |
| 279 | ABCG4_HUMAN | 347 | 356 | VPAPCPPCPP | 0.4 |
| 280 | TI17B_HUMAN | 160 | 169 | TPAPGYPSYQ | 0.4 |
| 281 | MLL4_HUMAN | 425 | 434 | PPPLCPPPPP | 0.4 |
| 282 | ACRO_HUMAN | 345 | 354 | PPPPSPPPPP | 0.5 |
| 283 | TCRG1_HUMAN | 101 | 110 | PPPMSSMPPP | 0.5 |
| 284 | FOXF2_HUMAN | 315 | 324 | SPVPSSPAMA | 0.5 |
| 285 | SALL3_HUMAN | 355 | 364 | APGLPSPLLP | 0.5 |
| 286 | GTR8_HUMAN | 43 | 52 | ALGYSSPAIP | 0.5 |
| 287 | ACK1_HUMAN | 795 | 804 | VPPGSSPLPP | 0.5 |
| 288 | ABI1_HUMAN | 392 | 401 | SPTPPPPPPP | 0.5 |
| 289 | WASF1_HUMAN | 321 | 330 | SPTPPPPPPP | 0.5 |
| 290 | ZN261_HUMAN | 815 | 824 | APTPPPPPPP | 0.5 |
| 291 | RGP1_HUMAN | 423 | 432 | APVLSSPPPA | 0.6 |
| 292 | ASPP2_HUMAN | 866 | 875 | YPPYPPPPYP | 0.6 |
| 293 | LIRB1_HUMAN | 635 | 644 | GPSPAVPSIY | 0.6 |
| 294 | PAR3L_HUMAN | 966 | 975 | ANVFRSPSPP | 0.6 |
| 295 | PDCD7_HUMAN | 34 | 43 | SPAFPPPLPQ | 0.6 |
| 296 | CCDC6_HUMAN | 440 | 449 | PPPPPPPPPM | 0.6 |
| 297 | SF01_HUMAN | 598 | 607 | APPPPPPPPM | 0.6 |
| 298 | TTC15_HUMAN | 242 | 251 | SPAPASPPPL | 0.6 |
| 299 | ABCCD_HUMAN | 130 | 139 | ILSFTSPLIM | 0.6 |
| 300 | NCOR2_HUMAN | 2003 | 2012 | APHHASPDPP | 0.6 |
| 301 | SORC1_HUMAN | 1127 | 1136 | LPSPPSPSTQ | 0.6 |
| 302 | UGDH_HUMAN | 42 | 51 | INAWNSPTLP | 0.6 |
| 303 | CX017_HUMAN | 102 | 111 | HPPLPPPPPP | 0.7 |
| 304 | DREB_HUMAN | 364 | 373 | PPPLPPPPPP | 0.7 |
| 305 | DTX1_HUMAN | 229 | 238 | APPLPPPPPP | 0.7 |
| 306 | PDE4D_HUMAN | 78 | 87 | PPPLPPPPPP | 0.7 |
| 307 | PRIMA_HUMAN | 57 | 66 | PPPLPPPPPP | 0.7 |
| 308 | SETBP_HUMAN | 1465 | 1474 | APPLPPPPPP | 0.7 |
| 309 | SETBP_HUMAN | 1473 | 1482 | PPPLPPPPPP | 0.7 |
| 310 | TNFL6_HUMAN | 51 | 60 | PPPLPPPPPP | 0.7 |
| 311 | VCIP1_HUMAN | 8 | 17 | PPPLPPPPPP | 0.7 |
| 312 | PO121_HUMAN | 749 | 758 | QPVFSSMGPP | 0.7 |
| 313 | GPNMB_HUMAN | 319 | 328 | APGPCPPPPP | 0.7 |
| 314 | OPLA_HUMAN | 1260 | 1269 | APPPGSPPQA | 0.7 |
| 315 | NGN1_HUMAN | 165 | 174 | LPPQCVPCLP | 0.7 |
| 316 | ULK1_HUMAN | 583 | 592 | SPPQASPPQP | 0.7 |
| 317 | ATRN_HUMAN | 315 | 324 | GPGCSVPVPA | 0.7 |
| 318 | EGFL4_HUMAN | 2070 | 2079 | PPAPPPPPPP | 0.7 |
| 319 | MAZ_HUMAN | 130 | 139 | PPAPPPPPPP | 0.7 |
| 320 | OST4_HUMAN | 4 | 13 | WPAPPPPPPP | 0.7 |
| 321 | RB_HUMAN | 20 | 29 | PPAPPPPPPP | 0.7 |
| 322 | SALL2_HUMAN | 157 | 166 | TPAPPPPPPP | 0.7 |
| 323 | SEM4G_HUMAN | 762 | 771 | APAPPPPPPP | 0.7 |
| 324 | TRRAP_HUMAN | 503 | 512 | VPAPPPPPPP | 0.7 |
| 325 | WASF2_HUMAN | 319 | 328 | PPAPPPPPPP | 0.7 |
| 326 | WASF4_HUMAN | 447 | 456 | PPAPPPPPPP | 0.7 |
| 327 | WT1_HUMAN | 57 | 66 | PPAPPPPPPP | 0.7 |
| 328 | NUMBL_HUMAN | 494 | 503 | YPGLGYPPMP | 0.7 |
| 329 | FEN1_HUMAN | 182 | 191 | CLTFGSPVLM | 0.8 |
| 330 | GIT1_HUMAN | 473 | 482 | QPPGPVPTPP | 0.8 |
| 331 | NKX25_HUMAN | 274 | 283 | YPAGPSPAQP | 0.8 |
| 332 | EP400_HUMAN | 96 | 105 | LPSPTSPGFQ | 0.8 |
| 333 | FYN_HUMAN | 38 | 47 | YPSFGVTSIP | 0.8 |
| 334 | IGHA1_HUMAN | 98 | 107 | TVPCPVPSTP | 0.8 |
| 335 | AMHR2_HUMAN | 123 | 132 | LPPPGSPGTP | 0.8 |
| 336 | TEAD1_HUMAN | 198 | 207 | APAPSVPAWQ | 0.8 |
| 337 | CAD16_HUMAN | 171 | 180 | APAQPSPDMF | 0.8 |
| 338 | FOXJ3_HUMAN | 564 | 573 | MPPPGYPHIP | 0.8 |
| 339 | SFR15_HUMAN | 715 | 724 | VPPPPPPPPF | 0.8 |
| 340 | WASF1_HUMAN | 462 | 471 | APGPHVPLMP | 0.8 |
| 341 | ZN295_HUMAN | 976 | 985 | PVPTNSPSPP | 0.9 |
| 342 | WASIP_HUMAN | 1 | 10 | MPVPPPPAPP | 0.9 |
| 343 | ZIC5_HUMAN | 134 | 143 | PPTPSPPPPP | 0.9 |
| 344 | SF3B4_HUMAN | 258 | 267 | PPAMPPPPMP | 0.9 |
| 345 | ZN261_HUMAN | 566 | 575 | VYQFCSPSCW | 0.9 |
| 346 | CT081_HUMAN | 321 | 330 | PMPFPYPLPQ | 0.9 |
| 347 | CAP1_HUMAN | 231 | 240 | PPPPGPPPPP | 0.9 |
| 348 | ENAH_HUMAN | 331 | 340 | PPPPGPPPPP | 0.9 |
| 349 | LTK_HUMAN | 33 | 42 | PLPLASPSPQ | 0.9 |
| 350 | UNC5B_HUMAN | 506 | 515 | LPPGTYPSDF | 0.9 |
| 351 | VASP_HUMAN | 170 | 179 | PPPPGPPPPP | 0.9 |
| 352 | VASP_HUMAN | 176 | 185 | PPPPGPPPPP | 0.9 |
| 353 | WASF2_HUMAN | 397 | 406 | PPPPGPPPPP | 0.9 |
| 354 | WASF3_HUMAN | 395 | 404 | PPPPGPPPPP | 0.9 |
| 355 | WASF4_HUMAN | 524 | 533 | PPPPGPPPPP | 0.9 |
| 356 | WASL_HUMAN | 380 | 389 | PPPPGPPPPP | 0.9 |
| 357 | WIRE_HUMAN | 8 | 17 | PPPPGPPPPP | 0.9 |
| 358 | CD2L7_HUMAN | 1269 | 1278 | PPGPPPPPPP | 0.9 |
| 359 | DDR1_HUMAN | 583 | 592 | GNTYAVPALP | 0.9 |
| 360 | GABR2_HUMAN | 9 | 18 | QPGPPPPPPP | 0.9 |
| 361 | HCN2_HUMAN | 19 | 28 | APGPPPPPPP | 0.9 |
| 362 | MMP24_HUMAN | 10 | 19 | APGPPPPPPP | 0.9 |
| 363 | DMRT2_HUMAN | 33 | 42 | TPPGPSPPPA | 1 |
| 364 | HCN4_HUMAN | 1026 | 1035 | SPPGHSPGPP | 1 |
| 365 | ZN198_HUMAN | 68 | 77 | PPPPSVPVVA | 1 |
| 366 | ATS19_HUMAN | 144 | 153 | PPPQPPPSPP | 1 |
| 367 | EP300_HUMAN | 861 | 870 | APVPTPPAMP | 1 |
| 368 | WASIP_HUMAN | 214 | 223 | SPGPTPPPFP | 1 |
| 369 | HXD8_HUMAN | 110 | 119 | PPPPHPPPPP | 1 |
| 370 | R3HDM_HUMAN | 460 | 469 | APPPHPPPPP | 1 |
| 371 | CAP1_HUMAN | 228 | 237 | GPPPPPPGPP | 1 |
| 372 | WASF2_HUMAN | 394 | 403 | PPPPPPPGPP | 1 |
| 373 | WASF3_HUMAN | 399 | 408 | GPPPPPPGPP | 1 |
| 374 | WASF4_HUMAN | 521 | 530 | PPPPPPPGPP | 1 |
| 375 | WASL_HUMAN | 377 | 386 | PPPPPPPGPP | 1 |
| 376 | WIRE_HUMAN | 5 | 14 | PPPPPPPGPP | 1 |
| 377 | ZN398_HUMAN | 279 | 288 | PVPFSSPPAA | 1 |
| 378 | SF3A1_HUMAN | 4 | 13 | GPVQAVPPPP | 1 |
| 379 | ASB3_HUMAN | 313 | 322 | VFGFSSPVCM | 1.1 |
| 380 | PDRN4_HUMAN | 351 | 360 | PPTPPVPDIC | 1.1 |
| 381 | SYNP2_HUMAN | 925 | 934 | YNPIHSPSYP | 1.1 |
| 382 | ZN179_HUMAN | 443 | 452 | GPGFTSPDEM | 1.1 |
| 383 | KPBB_HUMAN | 296 | 305 | LPCISYPAFA | 1.1 |
| 384 | CD4_HUMAN | 145 | 154 | SPPGSSPSVQ | 1.1 |
| 385 | CD5R2_HUMAN | 155 | 164 | PPPPPPPAPQ | 1.1 |
| 386 | DHX9_HUMAN | 80 | 89 | VPAFGVASPP | 1.1 |
| 387 | F8I2_HUMAN | 222 | 231 | LPPPPPPAPQ | 1.1 |
| 388 | ARHGB_HUMAN | 150 | 159 | SPPPPPPLPP | 1.1 |
| 389 | CAP2_HUMAN | 231 | 240 | LPPPPPPLPP | 1.1 |
| 390 | DAAM1_HUMAN | 562 | 571 | LPPPPPPLPP | 1.1 |
| 391 | DAAM2_HUMAN | 553 | 562 | PPPPPPPLPP | 1.1 |
| 392 | DOT1L_HUMAN | 1596 | 1605 | PPPPPPPLPP | 1.1 |
| 393 | DTX3_HUMAN | 138 | 147 | LPPPPPPLPP | 1.1 |
| 394 | ENAH_HUMAN | 312 | 321 | APPPPPPLPP | 1.1 |
| 395 | FA54A_HUMAN | 207 | 216 | SPPPPPPLPP | 1.1 |
| 396 | GBX2_HUMAN | 57 | 66 | PPPPPPPALP | 1.1 |
| 397 | PCLO_HUMAN | 2351 | 2360 | PPPPPPPLPP | 1.1 |
| 398 | PDE4D_HUMAN | 74 | 83 | QPPPPPPLPP | 1.1 |
| 399 | R3HDM_HUMAN | 471 | 480 | PPPPPPPLPP | 1.1 |
| 400 | RXRB_HUMAN | 142 | 151 | SPGLPPPAPP | 1.1 |
| 401 | SETBP_HUMAN | 1469 | 1478 | PPPPPPPLPP | 1.1 |
| 402 | SETBP_HUMAN | 1477 | 1486 | PPPPPPPLPP | 1.1 |
| 403 | SYNP2_HUMAN | 646 | 655 | PPPWPQPAPW | 1.1 |
| 404 | TNFL6_HUMAN | 47 | 56 | PPPPPPPLPP | 1.1 |
| 405 | TNFL6_HUMAN | 56 | 65 | PPPPPPPLPP | 1.1 |
| 406 | VCIP1_HUMAN | 4 | 13 | PPPPPPPLPP | 1.1 |
| 407 | WASF1_HUMAN | 425 | 434 | PPPPPPPLPP | 1.1 |
| 408 | Y0009_HUMAN | 168 | 177 | PPPPPPPLPP | 1.1 |
| 409 | YLPM1_HUMAN | 1353 | 1362 | PPPPPPPLPP | 1.1 |
| 410 | CT178_HUMAN | 189 | 198 | VPLPNVPSIA | 1.1 |
| 411 | RHG12_HUMAN | 257 | 266 | PPLPGSPAIQ | 1.1 |
| 412 | YN01_HUMAN | 149 | 158 | VPLPNVPSIA | 1.1 |
| 413 | S12A2_HUMAN | 34 | 43 | LPGTAVPSVP | 1.1 |
| 414 | PROL3_HUMAN | 42 | 51 | GPGFVPPPPP | 1.1 |
| 415 | NCOR2_HUMAN | 1046 | 1055 | PPCWTSGLPF | 1.1 |
| 416 | WASF2_HUMAN | 338 | 347 | PVGFGSPGTP | 1.2 |
| 417 | CADH8_HUMAN | 388 | 397 | PPVFSSPTYL | 1.2 |
| 418 | PCDH7_HUMAN | 140 | 149 | TPTFPSPVLT | 1.2 |
| 419 | PTC1_HUMAN | 642 | 651 | PPPYSSHSFA | 1.2 |
| 420 | LPHN1_HUMAN | 1411 | 1420 | PPPPAPPGPP | 1.2 |
| 421 | NR4A1_HUMAN | 194 | 203 | PPTGPSPSLA | 1.2 |
| 422 | TRIM8_HUMAN | 433 | 442 | QPVHSSPVFP | 1.2 |
| 423 | K1162_HUMAN | 183 | 192 | IPAWCSYVFF | 1.2 |
| 424 | PTN5_HUMAN | 35 | 44 | PPPPSPPSDP | 1.2 |
| 425 | WASF1_HUMAN | 350 | 359 | PPVPPPPPPP | 1.2 |
| 426 | FBLN4_HUMAN | 95 | 104 | GPPPPVPPAQ | 1.3 |
| 427 | NELFB_HUMAN | 569 | 578 | LPLPSVPAPA | 1.3 |
| 428 | TLN1_HUMAN | 724 | 733 | APTISSPVCQ | 1.3 |
| 429 | TLN2_HUMAN | 727 | 736 | SPTISSPVCQ | 1.3 |
| 430 | ACINU_HUMAN | 1118 | 1127 | HPPPPPPVQP | 1.3 |
| 431 | CN032_HUMAN | 79 | 88 | GPPPGPPAPF | 1.3 |
| 432 | HXB2_HUMAN | 75 | 84 | GPALPPPPPP | 1.3 |
| 433 | WBS14_HUMAN | 412 | 421 | PPPFPPMAPP | 1.3 |
| 434 | SHAN1_HUMAN | 1600 | 1609 | TPLPPVPPPA | 1.3 |
| 435 | CDX2_HUMAN | 259 | 268 | QPPPPPPQPP | 1.3 |
| 436 | KLH17_HUMAN | 26 | 35 | APPPPPPQPP | 1.3 |
| 437 | NCOR1_HUMAN | 1592 | 1601 | SPTPGYPSQY | 1.3 |
| 438 | PAX7_HUMAN | 471 | 480 | LVPWASPVPI | 1.3 |
| 439 | TCF20_HUMAN | 1555 | 1564 | QPPPPPPQPP | 1.3 |
| 440 | NAF1_HUMAN | 547 | 556 | AYPYAYPPMP | 1.3 |
| 441 | PLAL1_HUMAN | 236 | 245 | SVSPGSPPPP | 1.3 |
| 442 | ZIC5_HUMAN | 126 | 135 | PPPPAPPLPP | 1.3 |
| 443 | APOB_HUMAN | 3248 | 3257 | IPGYTVPVVN | 1.3 |
| 444 | ADAM8_HUMAN | 758 | 767 | SPPFPVPVYT | 1.3 |
| 445 | GSHI_HUMAN | 28 | 37 | LFPYAVPPPH | 1.3 |
| 446 | RXRB_HUMAN | 95 | 104 | VPPPSPPGPP | 1.3 |
| 447 | NLGN2_HUMAN | 798 | 807 | PPPPPPPSLH | 1.4 |
| 448 | 3BP1_HUMAN | 641 | 650 | SPSPASPGPA | 1.4 |
| 449 | BRPF1_HUMAN | 508 | 517 | APVVSVPCIP | 1.4 |
| 450 | PTN21_HUMAN | 515 | 524 | SYSFHSPSPY | 1.4 |
| 451 | A4GAT_HUMAN | 61 | 70 | CPTLTPPTPP | 1.4 |
| 452 | LIPA2_HUMAN | 961 | 970 | MVSLTSPSAP | 1.4 |
| 453 | LIPA3_HUMAN | 901 | 910 | MVSLTSPSAP | 1.4 |
| 454 | LIPA4_HUMAN | 408 | 417 | MVSLTSPSAP | 1.4 |
| 455 | PME17_HUMAN | 542 | 551 | QPVLPSPACQ | 1.4 |
| 456 | LAP4_HUMAN | 952 | 961 | GPLPPSPLPH | 1.4 |
| 457 | DGKD_HUMAN | 12 | 21 | PPQPPPPPPP | 1.4 |
| 458 | HD_HUMAN | 66 | 75 | QPQPPPPPPP | 1.4 |
| 459 | OST3B_HUMAN | 19 | 28 | LPQPPPPPPP | 1.4 |
| 460 | RFX1_HUMAN | 26 | 35 | QPQPPPPPPP | 1.4 |
| 461 | LEG4_HUMAN | 185 | 194 | PPTFNPPVPY | 1.4 |
| 462 | SMCA2_HUMAN | 298 | 307 | VPGPSVPQPA | 1.4 |
| 463 | BRD4_HUMAN | 973 | 982 | QPPPPPPPQP | 1.4 |
| 464 | PCBP4_HUMAN | 367 | 376 | ALPPASPGPP | 1.4 |
| 465 | NCOA6_HUMAN | 1746 | 1755 | PPCTSSPVVP | 1.5 |
| 466 | RBM9_HUMAN | 265 | 274 | VPGFPYPTAA | 1.5 |
| 467 | SLIK4_HUMAN | 588 | 597 | SAPFTSPAPA | 1.5 |
| 468 | TRRAP_HUMAN | 501 | 510 | APVPAPPPPP | 1.5 |
| 469 | BAI1_HUMAN | 1414 | 1423 | PPPPPPPPPQ | 1.5 |
| 470 | CECR6_HUMAN | 540 | 549 | APPPPPPPPQ | 1.5 |
| 471 | HD_HUMAN | 43 | 52 | PPPPPPPPPQ | 1.5 |
| 472 | MCR_HUMAN | 693 | 702 | QPPPPPPPPQ | 1.5 |
| 473 | TOB1_HUMAN | 248 | 257 | QPPPPPPPPQ | 1.5 |
| 474 | ATBF1_HUMAN | 2043 | 2052 | PPPPPPPPLP | 1.5 |
| 475 | CAP2_HUMAN | 228 | 237 | GPGLPPPPPP | 1.5 |
| 476 | DAAM1_HUMAN | 549 | 558 | LPPPPPPPLP | 1.5 |
| 477 | DAAM2_HUMAN | 552 | 561 | CPPPPPPPLP | 1.5 |
| 478 | DIAP1_HUMAN | 604 | 613 | PPPPPPPPLP | 1.5 |
| 479 | DIAP3_HUMAN | 334 | 343 | PPPPPPPPLP | 1.5 |
| 480 | DOT1L_HUMAN | 1595 | 1604 | PPPPPPPPLP | 1.5 |
| 481 | ENAH_HUMAN | 348 | 357 | PPPPPPPPLP | 1.5 |
| 482 | FOSB_HUMAN | 256 | 265 | LPPPPPPPLP | 1.5 |
| 483 | HXB2_HUMAN | 78 | 87 | LPPPPPPPLP | 1.5 |
| 484 | PCLO_HUMAN | 2350 | 2359 | PPPPPPPPLP | 1.5 |
| 485 | R3HDM_HUMAN | 470 | 479 | PPPPPPPPLP | 1.5 |
| 486 | RBM15_HUMAN | 315 | 324 | LPPPPPPPLP | 1.5 |
| 487 | SETBP_HUMAN | 1468 | 1477 | LPPPPPPPLP | 1.5 |
| 488 | SETBP_HUMAN | 1476 | 1485 | LPPPPPPPLP | 1.5 |
| 489 | TBX5_HUMAN | 306 | 315 | PPPNPYPLPQ | 1.5 |
| 490 | TNFL6_HUMAN | 46 | 55 | PPPPPPPPLP | 1.5 |
| 491 | TNFL6_HUMAN | 55 | 64 | PPPPPPPPLP | 1.5 |
| 492 | VCIP1_HUMAN | 3 | 12 | QPPPPPPPLP | 1.5 |
| 493 | WASF1_HUMAN | 323 | 332 | TPPPPPPPLP | 1.5 |
| 494 | WASF1_HUMAN | 424 | 433 | PPPPPPPPLP | 1.5 |
| 495 | Y0009_HUMAN | 167 | 176 | HPPPPPPPLP | 1.5 |
| 496 | YLPM1_HUMAN | 1352 | 1361 | PPPPPPPPLP | 1.5 |
| 497 | ANX11_HUMAN | 165 | 174 | QPVPSYPGYP | 1.5 |
| 498 | CNTN5_HUMAN | 213 | 222 | VVLMCSPPPH | 1.5 |
| 499 | RRA_HUMAN | 72 | 81 | VPSPPSPPPL | 1.5 |
| 500 | TAF1_HUMAN | 157 | 166 | PPPPPPPGPM | 1.5 |
| 501 | TAF1L_HUMAN | 156 | 165 | PPPPPPPGPM | 1.5 |
| 502 | 3BP2_HUMAN | 201 | 210 | PPAYPPPPVP | 1.5 |
| 503 | TAF4_HUMAN | 269 | 278 | APPPPPPAPA | 1.5 |
| 504 | TCRG1_HUMAN | 260 | 269 | TPTTSSPAPA | 1.5 |
| 505 | ANDR_HUMAN | 371 | 380 | GPPPPPPPPH | 1.5 |
| 506 | KRA42_HUMAN | 57 | 66 | QPTCCSPSCC | 1.5 |
| 507 | SMN_HUMAN | 218 | 227 | PPPPPPPPPH | 1.5 |
| 508 | WASL_HUMAN | 286 | 295 | GPPPPPPPPH | 1.5 |
| 509 | WT1_HUMAN | 60 | 69 | PPPPPPPPPH | 1.5 |
| 510 | HCN2_HUMAN | 17 | 26 | TPAPGPPPPP | 1.5 |
| 511 | ZN261_HUMAN | 891 | 900 | SMPIPVPVPM | 1.5 |
| 512 | WNK2_HUMAN | 846 | 855 | PLPPASPALP | 1.5 |
| 513 | CAP2_HUMAN | 235 | 244 | PPPLPPPGPP | 1.6 |
| 514 | ITPR2_HUMAN | 938 | 947 | IFPMSVPDVP | 1.6 |
| 515 | P2RY2_HUMAN | 322 | 331 | PPTGPSPATP | 1.6 |
| 516 | ANX11_HUMAN | 147 | 156 | QPPVTYPGQP | 1.6 |
| 517 | ACROL_HUMAN | 163 | 172 | PPPPPPPSPL | 1.6 |
| 518 | SEM5A_HUMAN | 598 | 607 | WTPWTSWSPC | 1.6 |
| 519 | FMR2_HUMAN | 880 | 889 | LPPCISPAPP | 1.6 |
| 520 | DLX4_HUMAN | 301 | 310 | SVSPCSPPLP | 1.6 |
| 521 | DTX3_HUMAN | 142 | 151 | PPPLPPPLPP | 1.6 |
| 522 | DAZP1_HUMAN | 295 | 304 | GPPPPPPDQF | 1.6 |
| 523 | CBP_HUMAN | 1942 | 1951 | VPAPPPPAQP | 1.7 |
| 524 | FA43B_HUMAN | 305 | 314 | APAPPPPAQP | 1.7 |
| 525 | PCDH9_HUMAN | 139 | 148 | APMFPSPVIN | 1.7 |
| 526 | BAT2_HUMAN | 661 | 670 | PPTPVPPSPP | 1.7 |
| 527 | CD5R2_HUMAN | 123 | 132 | VPVPTVPAAA | 1.7 |
| 528 | SIN3B_HUMAN | 724 | 733 | QPPLPPPAPH | 1.7 |
| 529 | CPSF3_HUMAN | 323 | 332 | SVVMASPGMM | 1.7 |
| 530 | MEA1_HUMAN | 137 | 146 | LPAPGVPAWA | 1.7 |
| 531 | COH1_HUMAN | 752 | 761 | SYCLPVPVIP | 1.7 |
| 532 | CTR4_HUMAN | 422 | 431 | SPGPASPGPL | 1.7 |
| 533 | PLS1_HUMAN | 41 | 50 | GPQVSYPPPP | 1.7 |
| 534 | COFA1_HUMAN | 149 | 158 | APAFSVPVMT | 1.7 |
| 535 | EPHB6_HUMAN | 312 | 321 | GNAPCSPCPA | 1.7 |
| 536 | RBM12_HUMAN | 209 | 218 | PVPPPVPTLP | 1.7 |
| 537 | SEM4C_HUMAN | 2 | 11 | APHWAVWLLA | 1.7 |
| 538 | SYNP2_HUMAN | 1027 | 1036 | VPAYTSPPSF | 1.7 |
| 539 | HME1_HUMAN | 53 | 62 | QPAPPSPPAA | 1.7 |
| 540 | MGR5_HUMAN | 1160 | 1169 | LVALTPPSPF | 1.7 |
| 541 | OPRD_HUMAN | 199 | 208 | MLQFPSPSWY | 1.7 |
| 542 | COTE1_HUMAN | 478 | 487 | PPHPASPPPL | 1.8 |
| 543 | PDCD7_HUMAN | 16 | 25 | PPQPPPPAPF | 1.8 |
| 544 | RBM12_HUMAN | 205 | 214 | IPPIPVPPPV | 1.8 |
| 545 | MEF2B_HUMAN | 134 | 143 | APAMPSPDVV | 1.8 |
| 546 | MUC5B_HUMAN | 5207 | 5216 | TPPTASPAAP | 1.8 |
| 547 | NFIX_HUMAN | 461 | 470 | ALTPPSPSFA | 1.8 |
| 548 | PLSB_HUMAN | 558 | 567 | TPSTTVPSVF | 1.8 |
| 549 | ABI2_HUMAN | 273 | 282 | VPTPSPPSVF | 1.8 |
| 550 | BHLH3_HUMAN | 213 | 222 | PLAYCVPVIQ | 1.8 |
| 551 | IF4G3_HUMAN | 53 | 62 | HLPMPYPVPQ | 1.8 |
| 552 | ATF5_HUMAN | 122 | 131 | LPPPSPPPLP | 1.8 |
| 553 | IRX3_HUMAN | 316 | 325 | PVAVASPSLP | 1.8 |
| 554 | ACRO_HUMAN | 347 | 356 | PPSPPPPPPP | 1.8 |
| 555 | ACROL_HUMAN | 158 | 167 | PPSPPPPPPP | 1.8 |
| 556 | DAAM2_HUMAN | 540 | 549 | LPPPPPPLPF | 1.8 |
| 557 | FOSB_HUMAN | 257 | 266 | PPPPPPPLPF | 1.8 |
| 558 | GEMI5_HUMAN | 475 | 484 | TLAWGPPVPP | 1.8 |
| 559 | KLF4_HUMAN | 334 | 343 | HPGPNYPSFL | 1.8 |
| 560 | ZIC5_HUMAN | 136 | 145 | TPSPPPPPPP | 1.8 |
| 561 | RW1_HUMAN | 1225 | 1234 | QPPLPPPVPQ | 1.8 |
| 562 | CT177_HUMAN | 103 | 112 | IPIPPSPLTP | 1.8 |
| 563 | EVL_HUMAN | 182 | 191 | PPPPPPPPVP | 1.8 |
| 564 | RHBT2_HUMAN | 240 | 249 | PPIIVVPDPP | 1.8 |
| 565 | RGL2_HUMAN | 589 | 598 | SPSLHSPADP | 1.8 |
| 566 | TULP4_HUMAN | 852 | 861 | PPPLPPPQPP | 1.8 |
| 567 | RNC_HUMAN | 114 | 123 | PPCFPPMPPP | 1.9 |
| 568 | WIRE_HUMAN | 1 | 10 | MPIPPPPPPP | 1.9 |
| 569 | YLPM1_HUMAN | 1349 | 1358 | VPIPPPPPPP | 1.9 |
| 570 | HXD8_HUMAN | 112 | 121 | PPHPPPPPPP | 1.9 |
| 571 | NFC4_HUMAN | 802 | 811 | GLPFSPPAPF | 1.9 |
| 572 | NGAP_HUMAN | 483 | 492 | CPAIMSPSLF | 1.9 |
| 573 | R3HDM_HUMAN | 462 | 471 | PPHPPPPPPP | 1.9 |
| 574 | SYGP1_HUMAN | 584 | 593 | CPAIMSPSLF | 1.9 |
| 575 | Y0009_HUMAN | 165 | 174 | PPHPPPPPPP | 1.9 |
| 576 | ACRO_HUMAN | 349 | 358 | SPPPPPPPPA | 1.9 |
| 577 | BAT3_HUMAN | 660 | 669 | PPPPPPPPPA | 1.9 |
| 578 | CEBPB_HUMAN | 162 | 171 | PPPPPPPPPA | 1.9 |
| 579 | ENAH_HUMAN | 360 | 369 | VPPPPPPPPA | 1.9 |
| 580 | GABR2_HUMAN | 12 | 21 | PPPPPPPPPA | 1.9 |
| 581 | GBX2_HUMAN | 55 | 64 | LPPPPPPPPA | 1.9 |
| 582 | KIF5C_HUMAN | 300 | 309 | IVICCSPSVF | 1.9 |
| 583 | RANB9_HUMAN | 84 | 93 | PPPPPPPPPA | 1.9 |
| 584 | RBL2_HUMAN | 8 | 17 | SPPPPPPPPA | 1.9 |
| 585 | RBM12_HUMAN | 180 | 189 | SPMNTVPPPP | 1.9 |
| 586 | SEM4G_HUMAN | 766 | 775 | PPPPPPPPPA | 1.9 |
| 587 | TRRAP_HUMAN | 507 | 516 | PPPPPPPPPA | 1.9 |
| 588 | UBP51_HUMAN | 132 | 141 | PPPPPPPPPA | 1.9 |
| 589 | WASF2_HUMAN | 363 | 372 | APPPPPPPPA | 1.9 |
| 590 | ZIC5_HUMAN | 141 | 150 | PPPPPPPPPA | 1.9 |
| 591 | ZIC5_HUMAN | 392 | 401 | PPPPPPPPPA | 1.9 |
| 592 | ANXA7_HUMAN | 6 | 15 | YPPTGYPPFP | 1.9 |
| 593 | LRP2_HUMAN | 4611 | 4620 | ATPPPSPSLP | 1.9 |
| 594 | SPIB_HUMAN | 48 | 57 | TVAPPVPATP | 1.9 |
| 595 | IRX1_HUMAN | 406 | 415 | LPAPPPPQPP | 1.9 |
| 596 | EP400_HUMAN | 32 | 41 | PNPPPSPAAP | 1.9 |
| 597 | MUC13_HUMAN | 63 | 72 | FPTATSPAPP | 1.9 |
| 598 | EPN1_HUMAN | 511 | 520 | SPVPPVPGAP | 1.9 |
| 599 | DOCK1_HUMAN | 1823 | 1832 | SPTPPPPPPH | 1.9 |
| 600 | PCLO_HUMAN | 3818 | 3827 | QYQYSSPALP | 1.9 |
